# Advanced Strategies to Improve Performances of Molybdenum-Based Gas Sensors

**DOI:** 10.1007/s40820-021-00724-1

**Published:** 2021-10-11

**Authors:** Angga Hermawan, Ni Luh Wulan Septiani, Ardiansyah Taufik, Brian Yuliarto, Suyatman, Shu Yin

**Affiliations:** 1 Faculty of Textile Science and Engineering, Shinshu University, 3-15-1 Tokida, Ueda, Nagano, 386-8567 Japan; 2 Advanced Functional Materials Research Group, Institut Teknologi Bandung, Bandung, 40132 Indonesia; 3 Research Center for Nanosciences and Nanotechnology (RCNN), Institut Teknologi Bandung, Bandung, 40132 Indonesia; 4 Institute of Multidisciplinary Research for Advanced Material (IMRAM), Tohoku University, 2-1-1 Katahira, Aoba-ku, Sendai, Miyagi 980-8577 Japan

**Keywords:** Molybdenum based, MoO_3_, MoS_2_, Gas sensing, Advanced strategy

## Abstract

Molybdenum-based materials have been intensively investigated for high-performance gas sensor applications. Particularly, molybdenum oxides and dichalcogenides nanostructures have been widely examined due to their tunable structural and physicochemical properties that meet sensor requirements. These materials have good durability, are naturally abundant, low cost, and have facile preparation, allowing scalable fabrication to fulfill the growing demand of susceptible sensor devices. Significant advances have been made in recent decades to design and fabricate various molybdenum oxides- and dichalcogenides-based sensing materials, though it is still challenging to achieve high performances. Therefore, many experimental and theoretical investigations have been devoted to exploring suitable approaches which can significantly enhance their gas sensing properties. This review comprehensively examines recent advanced strategies to improve the nanostructured molybdenum-based material performance for detecting harmful pollutants, dangerous gases, or even exhaled breath monitoring. The summary and future challenges to advance their gas sensing performances will also be presented.

## Highlights

- Various advanced strategies for improving gas sensing performances of molybdenum-based nanostructures are reviewed.
- The plausible mechanism of enhanced gas sensing properties from each strategy is discussed.
- The conclusive outlook, challenge, and suggestions for future development toward marked commercialization of molybdenum-based gas sensing devices are provided.

## Graphical abstract

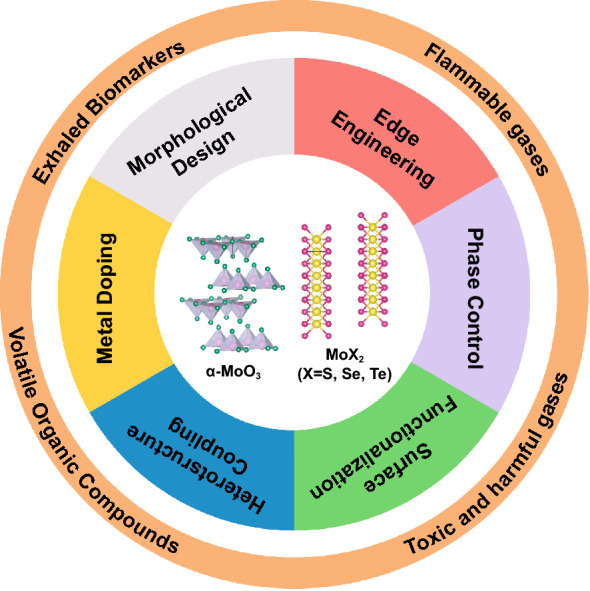

## Introduction

With advancements in technology, science, and economic mobilities, pollution has become a global concern, especially emissions from vehicles, various industrial processes and transports, agriculture, and residential activities [1–6]. For instance, air pollutions contain many particulate matters and harmful gases directly impacting the environment and human beings [1, 2]. The emissions mostly contain NO_x_, CO, SO_2_, NH_3_, and volatile organic compounds (VOCs) [5], which cause global warming and climate change. Human health is also at risk because these toxic gases enter undetectably (because some gases have colorless and odorless properties) to the body through oral intake, inhalation, and skin contact, causing serious problems that might eventually lead to death [1, 2, 7–9]. According to the World Health Organization (WHO), air pollution is responsible for nearly 800,000 premature deaths per year [10]. Some gases, such as H_2_, propane, and methane, are highly explosive without proper handling and safety measures. Therefore, there is a need to develop advanced gas sensor devices to detect these deleterious, dangerous and poisonous pollutants and reduce their damaging effect [4, 11]. Moreover, existing gas sensing technology is forecasted to take a dominant role in health monitoring and disease prediction *by* analyzing exhaled breath biomarkers [12–14].

Gas sensor devices, based on their working mechanism, are mainly classified into chemoresistive, electrochemical, optical, surface acoustic, surface plasmon resonance, and micro-cantilever sensors [15–21]. Among them, the chemoresistive-type gas sensor is the most popular due to its low cost, high sensitivity, fabrication simplicity, ease of miniaturization, and portability, apart from having a well-accepted empirically gas sensing mechanism [22, 23]. The term chemoresistive originates from its working principle in which sensing measurement is based on the change in electrical resistivity upon target gas or chemical exposure. Therefore, the active sensing materials should possess distinguished electrical properties in the different surrounding atmospheres. Metal semiconductors are generally utilized as active materials to sense gases. Initially, the gas sensing materials in sensor device are exposed to the air atmosphere at certain temperatures based on their optimum working conditions. The oxygen molecules (O_2_) are then adsorbed onto the surface of materials by catching electrons near the conduction band, creating electron depletion layers (EDLs) in n-type semiconductor and hole accumulation layers (HALs) in p-type semiconductor materials. The adsorbed oxygen transforms into different ion species O^2−^, O^−^ and O^2−^ [24, 25]. Due to the charge carrier concentration difference in the material before and after exposure in ambient, the internal resistance is altered.

At this point, the measured resistance represents sensor resistance in the air (*R*_*a*_) [26, 27]. When the target gas flows and comes into the sensor system, the sensor resistance changes due to the active reaction between ionized oxygen, releasing trapped electrons from the depleted region [27, 28]. The measured resistance in the sensor represents sensor resistance in the air (*R*_*g*_). Depending on the nature of semiconducting materials, the sensor sensitivity (*S*) can be calculated by *R*_*a*_/*R*_*g*_ for n-type and *R*_*g*_/*R*_*a*_ for p-type. Sometimes when the resistance difference is too small, sensitivity is defined as the relative change in resistance or *S* = (*R*_*a*_-*R*_*g*_/*R*_*g*_) × 100% for n-type and *S* = (*R*_*g*_-*R*_*a*_/*R*_*a*_) × 100% for p-type [29]. According to this mechanism, the high sensitivity value is an important parameter for gas sensing materials. Furthermore, operating temperatures, selectivity, response–recovery times, long-term stability, and durability against extreme conditions are crucial for evaluating gas sensor device performances [30].

For decades, studies have been conducted on the potentiality of various types of semiconducting materials for an active component in chemoresistive gas sensors, including metal oxides, sulfides/oxysulfides, nitrides/oxynitrides, and fluoride/oxyfluorides, as well as optimizing their gas sensing properties through advanced strategies [30–34]. Considerable efforts have also been directed toward the investigation of a different class of materials, including molybdenum-based gas sensing materials, which are an attractive group of materials for a wide range of applications, including catalyst, photocatalyst, gas sensor, biomedical therapy, energy storage and conversion, and optoelectronic devices owing to unique tunability of physical and chemical characteristic [32, 35–41]. The most important materials in this group are alpha-molybdenum oxide (α-MoO_3_) and molybdenum sulfide (MoS_2_). These two are promising candidates for high-performance gas sensor applications because their unique layered 2D structures allow gaseous compounds to access more adsorption sites. This is where the adsorption/desorption process extensively occurs, leading to high sensitivity [32, 42]. With a high aspect ratio, 2D-structured α-MoO_3_ and MoS_2_ naturally exhibit high specific surface area that is undoubtedly beneficial for gas adsorption [43]. The synergistic effect of physical, electronic, chemical, and mechanical properties was previously examined for *α*-MoO_3_- and MoS_2_-based sensing materials. Furthermore, enormous research strategies have been employed through morphology and crystal phase control, facet engineering, surface functionalization with noble metals, elemental doping, and heterostructures coupling to escalate their gas sensing performance and meet the expected criteria for mass productions. Some previous reviews have been published elsewhere, but they focused on general synthesis and applications of molybdenum-based materials [37, 44]. No recent report has focused on advanced strategies for optimization of their gas sensing performance.

This review provides a comprehensive perspective of α-MoO_3_ and MoS_2_ as gas sensing materials. The basic crystal structures of these materials and their common properties include physical, electrical, electronics, optical, chemical, and mechanical that strongly correlate to their gas sensing behavior are presented. Afterward, the focus is on the most recent and advanced strategies to optimize gas sensing performances of α-MoO_3_ and MoS_2_ in detecting various harmful gases. It is noteworthy that the recent progress on the gas sensing performance of other molybdenum-based materials, such as MoSe_2_, MoTe_2_, Mo_2_C, and MoC, is briefly discussed to encourage further extensive development. This review also summarizes molybdenum-based gas sensing materials and an overview, including challenges and future works.

## Molybdenum Oxide (MoO_3_) Gas Sensing Materials

**Fig. 1:**
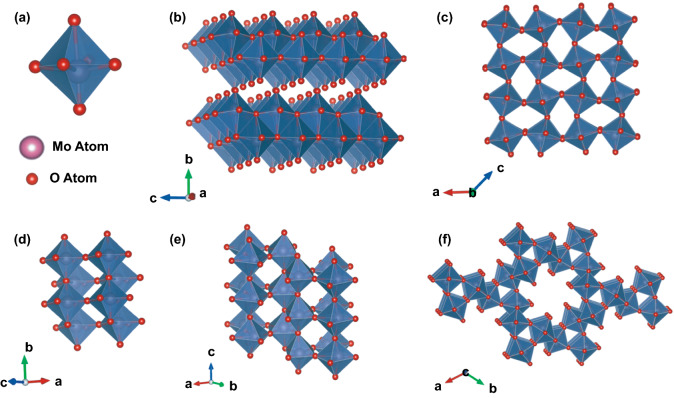
**a** MoO_6_ octahedra in the thermodynamically stable *α*-MoO_3_ phases. **b** Orthorhombic *α*-MoO_3_ with a layered structure. **c** Metastable monoclinic *β*-MoO_3_. **d** *ε*-MoO_3_, also known as MoO_3_-II. **e** Metastable h-MoO_3_. **f** Tunnel structure along the c-axis of h-MoO_3_ unit cell. Reproduced with permission from Ref. [43]. Copyright 2017 Wiley-VCH

Molybdenum oxide (MoO_3_) is one of the n-type metal oxide semiconductors with a band gap ranging from 2.39 to 2.9 eV [45–47]. This oxide has unique optical and electronic properties, layered structure, and good catalytic properties suitable for photodevice, energy storage, and catalyst [43]. Furthermore, its intrinsic semiconductor property with high sensitivity to the presence of gas explains its wide use as a gas sensor material [43, 48]. Regarding crystal structure, MoO_3_ exist in three different types of structures depending on growth temperature, pressure, and impurities [43]. The structures are orthorhombic (*α*-MoO_3_), monoclinic (*β*-MoO_3_), hexagonal (*h*-MoO_3_), and *ε*-MoO_3_, as shown in Fig. 1. However, *α*-MoO_3_ is the most popular and widely used since it is stable thermodynamically and often formed at high temperatures. In this type of structure, the distorted MoO_6_ octahedral are arranged in layers toward the *b* axis with corner and edge-sharing [49]. This layered structure is supported by Mo–O's asymmetry coordination, where the distance between them is varied from 1.67 to 2.33 Å [50]. In the gas sensor application, this phase is popular due to its high stability. The other phases, *β*-MoO_3_ and *h*-MoO_3,_ are metastable and need a complex preparation procedure to prepare [51]. In general, *β*-MoO_3_ is prepared by the cation exchange approach, while *h*-MoO_3_ preparation involves alkaline earth metal [52]. Despite the superior nature of the catalyst, *β*-MoO_3_ is a rarely found in gas sensor application. Regarding *h*-MoO_3_, several studies have utilized this phase to detect ethanol, formaldehyde, acetone, NH_3_, and H_2_. In terms of electronic properties, the n-type semiconductor properties of MoO_3_ are supported by the presence of oxygen vacancies, which induce localization of electrons on the surface [53]. These electrons fill the 4d state of Mo in the Mo–Mo bond, and the state is located in the MoO_3_ band gap. However, the number of oxygen vacancies strongly determines the electronic properties of MoOx and according to previous studies, MoO_2_ has metallic properties [53, 54]. In this section, the use of MoO_3_ semiconductors as gas sensors is reviewed. The development of MoO_3_ modifications to improve its performance as a gas sensor, such as morphology modification, metal decorated, elemental doping, and heterostructures, are also discussed in detail.

**Fig. 2:**
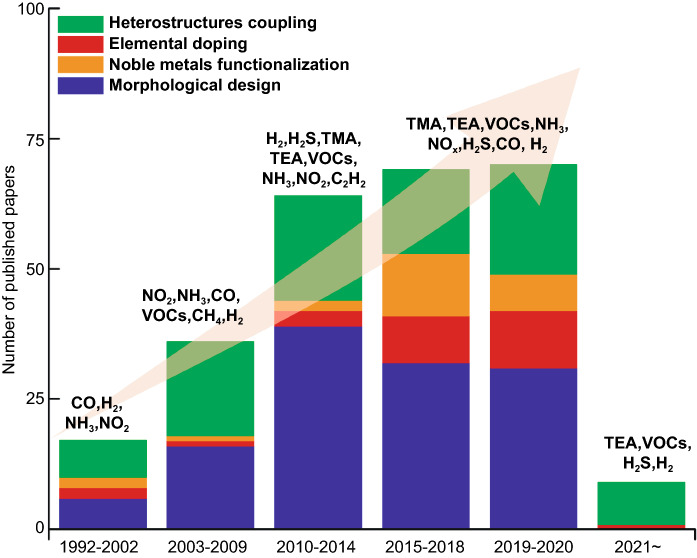
Number of publications examining various strategies to improve gas sensing properties of MoO_3_ toward wide range of gases. Data are collected from Web of Science (WoS) as of April 2, 2021, with the keyword “MoO_3_ gas sensing.” Both experimental and computational works are included. Review and perspective articles are excluded from the collected data. TMA and TEA stand for trimethylamine and triethylamine, respectively

From the literature research obtained from the Web of Science database as shown in Fig. 2, we discovered the total number of publications related to MoO_3_ based gas sensor is 265, with the first report of MoO_3_ was published in 1992. After a decade, the number of work is still few which the most of the work focused on thin-film preparation. The number of detected gas is limited to non-VOCs gas. Early investigations demonstrated MoO_3_ thin-film deposition method in electronic substrate via physical sputtering. Also, the intercalation of polymeric materials guest on interlayer MoO_3_ host became a major approach for increasing MoO_3_ gas sensing properties. Because of the development of sol–gel chemistry as a novel wet preparation of inorganic solid, various morphological nanostructured MoO_3_ such as nanoparticles, nanobelts, and nanoplatelets have been successfully synthesized within the time frame of 2010–2014. This approach attracted many researchers in the gas sensing field, as demonstrated by the number of publications that exceeded other approaches. We have recognized that the acidity of MoO_3_ is effective in detecting gases with basic nature, such as the gas with an amine group (TMA and TEA). The noble metals functionalization and elemental doping strategies have also been getting more popularity in recent years and are predicted to compete with the other 2 approaches. We will be discussing the development of each strategy in the following section.

### Morphological Design

The morphology design is essential in improving gas sensor performance because it strongly determines the active sites for surface reaction. Several efforts to design MoO_3_ from zero dimensional to hierarchical three dimensional have been recently reported. Each dimension has its role in enhancing sensor performances. Zero-dimensional nanomaterials commonly referred as quantum dots (QDs), typically semiconductor materials with a size less than 10 nm. This nanoscale size of MoO_3_ allows their electrons to be depleted entirely by oxygens, thus significantly improve the sensitivity [55]. The quantum confinement that occurs in quantum dots also makes their bandgap tunable depending on their size. Based on its property, its sensing performance can be altered by altering its size [55–57]. Moreover, the reactivity of the QDs is considerably high due to the many defects or oxygen vacancies present on their surface, increasing active sites for oxygen adsorption. In general preparation of MoO_3_ or MoO_x_ QDs, exfoliation process of MoS_2_ or MoO_3_ precursor is involved in the presence of an oxidant such as H_2_O_2_ that act as an exfoliating agent [58–60]. When MoS_2_ precursor is dispersed in the aqueous solution containing H_2_O_2_, the oxidant provides the excess of oxygen that induces exfoliation and oxidation of Mo^4+^ to its higher oxidation state. It was also reported that short oxidation of an aggressive oxidant of KMnO_4_, followed by oxidation by H_2_O_2_ in hydrothermal conditions, will also produce high quality of MoO_x_ QDs [61]. Moreover, post-treatment, such as thermal exfoliation and surfactants addition, were reported to control the size and stabilize the QDs [59]. Those techniques produced the QDs with the size in the range of 2–6 nm. Aside from MoS_2_, MoO_3_ powder was also reported as a precursor for the formation of MoO_x_ QDs. The exfoliation of MoO_3_ can be realized by dispersing the oxide in organic solvents such as dimethyl sulfoxide (DMSO) and N-methyl-2-pyrrolidinone (NMP) with the help of ultraviolet (UV) light [60, 62]. Although many reports in MoO_x_ QDs are available, their exploitation as gas sensor materials cannot be found. Therefore, the research on this topic is still widely opened. The QDs can be present as supporting material for other oxides or carbon nanomaterials.

Unlike zero-dimensional MoO_3_, other dimensions of this oxide, such as one dimensional (1D), two dimensional (2D), and three dimensional (3D), were widely examined as sensitive materials for gas sensors. 1D MoO_3_ is a popular gas sensor due to its high surface-to-volume ratio, high exposed facet, and high chemical stability [63–65]. Controlling their diameter close to the Debye length *(λ*_*L*_*)* can give various conduction states improving the sensing performances significantly. Moreover, the back-to-back *Schottky* barrier can be generated by contacting two or more random oriented MoO_3_ [66, 67]. The synergistic effect between the wide depletion layer on the 1D surface and the back-to-back *Schottky* barrier is believed as a reason for their excellent performances. In the case of MoO_3_-based gas sensors, several 1D nanostructures have been reported, including nanowires, nanorods, and nanobelts. Self-assembly α-MoO_3_ nanowires on a flexible hydrophobic substrate for H_2_ sensing was reported by Luo et al*.* [48]. Since MoO_3_ tends to form 2D in its growing process, modifications of the synthesis method need to be carried out. The nanowire was firstly prepared by hydrothermal method at 260 °C for 96 h. The formation of orthorhombic phase *α*-MoO_3_ nanorod occurred with a diameter of ~ 300 nm and a length of ~ 1 mm. The authors define its sensing performance by sensitivity factor, *β*, (R_air_-R_gas_)/R_air_. At room temperature, the resulted α-MoO_3_ displayed a good response toward 1.5% of H_2_ with a sensitivity factor of 0.85. Moreover, the materials can detect the gas in 3 s and need only 2.7 s to recover with remarkable selectivity. The excellent performances of the *α*-MoO_3_ nanowire are caused by the Mo^5+^ species contained in the oxide. Since this species has a stronger bonding with adsorbed oxygen, it provides more active sites for gas sensor reactions. Other 1D nanorods of *α*-MoO_3_ were prepared by Cao et al*.* [68] using a similar method with the assistance of hydrochloric acid (HCl) and cetyltrimethylammonium bromide (CTAB) at 180–190 °C for 24 h. The process led to nanorod formation with a diameter and length in the range of 100–200 nm and 1–3 µm, respectively. The resulting nanorod shows a response of 35 toward 400 ppm ethanol at a relatively high optimal temperature of 350 °C.

VOCs sensor based on α-MoO_3_ nanobelts was prepared by Jiang et al. [69] and Mo et al*.* [70] using a similar method, hydrothermal. Both groups used ammonium heptamolybdate tetrahydrate as a Mo source with different acids. Nanobelts of *α*-MoO_3_ with a width of 200 nm and length of ~ 6 µm were produced by a hydrothermally heated Mo precursor solution containing HNO_3_ at 180 °C for 36 h. The nanobelts show a response of ~ 3 to 100 ppm of xylene at 206 °C with the response and recovery times of 7 and 87 s, respectively. The addition of HCl as a pH modulator and hydrothermal condition at 160 °C for 15 h resulted in nanobelts structure with a width of 180 nm. Mo et al. reported that the prepared oxide displayed good performance as an ethanol sensor at 300 °C with a 50–800 ppm detection range. At its optimal temperature, the oxide has a response and recovery times in the range of 10–40 and ~ 4–70 s, respectively. Interestingly, the sensing mechanism of α-MoO_3_ is mainly contributed by surface lattice oxygen. The ethanol target is oxidized by the oxygen lattice, causing electron transfer to the metallic core and producing oxygen vacancies. This phenomenon changes the oxide resistance that is used as a sensor signal.

Naturally, MoO_3_ with orthorhombic crystal structure or α-MoO_3_ has a double-layer structure [43]. This feature of *α*-MoO_3_ offers the easiness to produce 2D morphologies, including a thin layer via the exfoliation process. The 2D material itself is considered a promising class of materials due to its unique properties, the high surface area that provide a huge number of active sites, and the possibility for surface modification as needed [71, 72]. As sensitive materials for gas sensors, increasing its affinity to target gas combine with the high surface area leads to superior gas sensor performance. Moreover, in the *α*-MoO_3_ case, the distance between its layer provides an additional diffusion path for gases to reach accessible sites. Several works report on the exfoliation of bulk *α*-MoO_3_ to 2D structures, such as nanoflakes [72, 73] and nanosheets [74]. Generally, the exfoliation process is successfully executed with ultrasonication assistance in the mixture of ethanol/water medium. Ji et al*.* [74] reported that nanosheets of α-MoO_3_ could be produced via exfoliation in the water/ethanol mixture with the ratio of 50%. Another liquid, such as DMSO, DMF, and IPA, produces a many layers of nanoflake. The nanosheets have superior alcohol sensing performance compared to the nanoflakes, with a response value of 31 at 300 °C to 100 ppm of alcohol vapor. The nanosheets with a higher surface area than the nanoflakes provide more active sites for surface reaction. Rahman et al*.* [75] also performed exfoliation using a different route. The CVD method was used to deposit α-MoO_3_ on the substrate. This technique produces nonstoichiometry of nanoflakes of α-MoO_3-x_ with many Mo^5+^ and oxygen vacancies on its surfaces. Since oxygen vacancies are the main key in the sensing mechanism of layered α-MoO_3_, the more vacancy, the higher the performance will be. The nanoflakes show good performance to NO_2_ and H_2_S at 250 °C with excellent selectivity.

Surface modification by enriching oxygen vacancy on MoO_3_ surface for TMA sensing was carried out by Shen et al*.* [76] The ultrasonication of bulk MoO_3_ in the solution containing methanol and H_2_O_2_, followed by solvothermal at 180 °C for 12 h were performed to exfoliate the bulk oxide. The nanosheets with a thickness of 28 nm and rich in oxygen vacancies were obtained after calcining the solvothermal product at 400 °C. The abundance of oxygen vacancies provides many delocalized electrons that support charge transfer between the surface and TMA. These nanosheets detect 50 ppm of TMA at the optimal temperature of 133 °C with a response of 198. The different surface modifications with different results in types of gas sensor behavior were observed by Bisht et al. [77]. In their work, *α*-MoO_3_ was deposited using the pulsed laser deposition (PLD) technique on Si/SiO_2_ substrates. By varying the number of pulses, 2D, ultrathin-film (UTF) and thin-film (TF) α-MoO_3_ were produced with the thickness of 6, 18, and 80 nm, respectively. Interestingly only TF exhibits n-type behavior while 2D and UTF exhibit p-type behavior during NO_2_ exposure at 100 °C. Two reasons are believed to cause this unusual behavior of 2D and UTF; first, the high number of oxygen species on the surface of 2D and UTF induce the inversion layer resulting in the domination of holes in their conduction process. Second, the *Schottky* barriers at metal-*α*-MoO_3_ contact of 2D and UTF are higher than that of TF preventing the electrons transfer from metal to α-MoO_3_ and allowing holes to pass the conduction channel. At 100 °C, the 2D *α*-MoO_3_ shows the highest response to 10 ppm of NO_2_ with a response value of 25% and response time of 200 s.

**Fig. 3:**
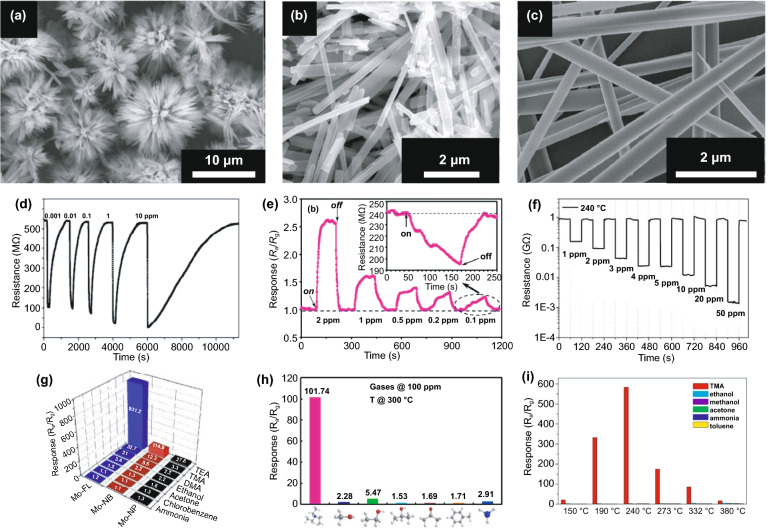
α-MoO_3_ flower-like showing superior TEA sensor performance at 170 °C (**a, d, g**) compared to α-MoO_3_ nanorod that has optimal temperature of 300 °C (**b, e, h**) and α-MoO_3_ nanobelts that have optimal temperature of 240 °C (**c, f, i**). (**a, d, g**) are reproduced with permission from Ref. [87]. Copyright 2015, The Royal Society of Chemistry. (**b**, **e**, **h**) are reproduced with permission from Ref. [78]. Copyright 2019, Elsevier. (**c**, **f**, **i**) are reproduced with permission from Ref. [26]. Copyright 2016, Elsevier

Several studies show that low dimensional of MoO_3,_ such as nanorods [78–80], nanowires [48, 81], nanobelts [82, 83], nanoflakes [26, 84], and nanosheets [45, 49, 85] have good performance as sensitive materials for toxic gas detection. However, some works reported that hierarchical 3D structures assembled by their low dimensional form offer higher performance due to their low density, high surface area, and porosity that allow more adsorption sites [86, 87]. Huo’s group compared the TEA sensor performance of *α*-MoO_3_ nanoparticles, nanobelts, and nanobelt-assembled hierarchical flower-like [87] at 170 °C. *α*-MoO_3_ flower-like show superior response of 931.2 to 10 ppm TEA, 8.1 and 33.7, higher than the value generated by nanobelts and nanoparticles, respectively. The high performance is attributed to the combination of high surface area and high (010) facet. Some studies also report the active facet of (010), especially to TEA [88, 89]. The flower-like *α*-MoO_3_ produced by Huo’s group also shows higher performance than ultralong *α*-MoO_3_ nanobelts and nanorod, which show the highest performance to detect TEA at 240 and 300 °C [78, 83], respectively, as shown in Fig. 3. The microboxes of α-MoO_3_ composed of nanosheets are obtained using MnCO_3_ microboxes as a template [90]. In this case, the template was removed by an acid treatment. As a gas sensor, the boxes show good performance in detecting 100 ppm ethanol at 260 °C with response value and response time of 78 and 15 s, respectively. Its performance is higher than other works that developed sponge-like nanorods, nanofibers, and nanobelts *α*-MoO_3_ [89, 91].

In the case of 3D *α*-MoO_3_, the gas sensor performances also depend on its assembly units. For instance, Ji et al*.* prepared a hierarchical 3D structure assembled from nanosheets with three different thicknesses, 65–80, 30–40, and 5–8 nm [85]. The gas sensor measurements to 300 ppm of ethanol at 300 °C show the thinnest sheets show the highest performances due to its relatively larger surface area. Furthermore, the two assembly units, nanofibers and nanosheets, assemble a 3D sphere of α-MoO_3_ were prepared by Ji et al*.* as ethanol sensors [92]. The nanosheets show a higher response to 400 ppm of ethanol at 300 °C than the nanofiber one due to their higher surface area and there are many intersections between individual sheets that force gas to adsorb on the surface effectively. However, the higher diffusion rate and lower potential energy of the nanofiber-assembled sphere lead to a faster response.

The morphology design apparently can reduce the optimal temperature which has an impact on increasing sensor stability. Efforts to reduce the working temperature of a pure MoO_3_-based gas sensor can be done by making the 2D structure as thin as possible. The thin 2D structure has abundant defects and oxygen vacancies providing a large amount of electron delocalization so that the reactivity increases at lower temperatures. However, another challenge in the utilization of this oxide is the negative effect of humidity. High humidity generally reduces sensor performance because moisture on the surface can hinder oxide and target gas interaction. Therefore, other efforts such as modification with noble metals, elemental doping, and creating heterostructures can minimize the influence of humidity.

### Surface Functionalization with Noble Metals

**Fig. 4:**
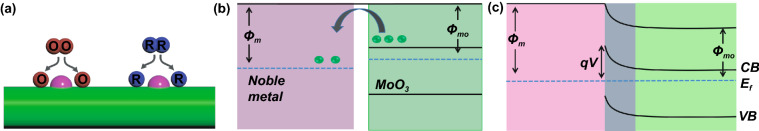
**a** Spill-over effect or chemical sensitization induced by noble metal on the surface of MoO_3_. **b, c** Electron transfer from MoO_3_ to noble metal right after making contact along with Fermi alignment that generates Schottky barrier at the interface of noble metal/MoO_3_

Improvement in the gas sensor performance can be achieved by introducing a noble metal on the metal oxide surface. Apart from their action as active sites, noble metals with high catalytic activity also reduce the activation energy of a gas, leading to an increase in adsorption rate and lowering operating temperature [93–95]. Improvement of sensor performance due to the functionalization of noble metal is attributed to its ability to induce electronic and chemical sensitization. Fermi level differences between noble metal and metal oxide generate a Schottky barrier at the interface that is sensitive to the presence of gas (electronic sensitization). For instance, some noble metals, such as Au [26, 45, 96–98], Ag [99], Pt [100], and Pd [101], were reported to enhance gas sensors based on MoO_3_. These metals have a higher work functions of 5.1 [97, 102], 4.72 [103], 5.6 [104], and 5.2 eV [105], respectively, compared to work function of MoO_3_ that is 2.9 eV [106]. These differences induce the occurrence of electronic sensitization. Moreover, the dispersion of noble metals on the surface of metal oxides induces spill-over effects that help to increase the rate of surface reactions, leading to reduce response and recovery times. This chemical sensitization also helps to convert unreactive gas into a reactive form and improve selectivity [107–110]. Figure 4 shows an illustration of chemical and electronic sensitization in noble metal decorated MoO_3_. Under an air atmosphere, the noble nanoparticles on MoO_3_ surface act as an active site for O_2_ dissociation. During gas exposure, for example R_2_, the noble metal dissociates them to R, which is more reactive than R_2_, as seen in Fig. 4a. This spill-over effect increases the reaction rate that resulting in reduced response time and lowered operating temperature. As mentioned earlier, electronic sensitization occurs due to the difference in work function between noble metal and MoO_3_. Almost all noble metals have a work function higher than that of MoO_3._ Right after MoO_3_ makes contact with noble metals, electrons will flow from MoO_3_ to noble metal along with Fermi level alignment leading to upward bending of MoO_3_ (Fig. 4b). The band bending is associated with barrier potential qV at the noble metal/MoO_3_ interface that can be changed during the surface reaction as shown in Fig. 4c. The combination of chemical and electronic sensitizations results in high sensitivity, low temperature, and fast response.

**Fig. 5:**
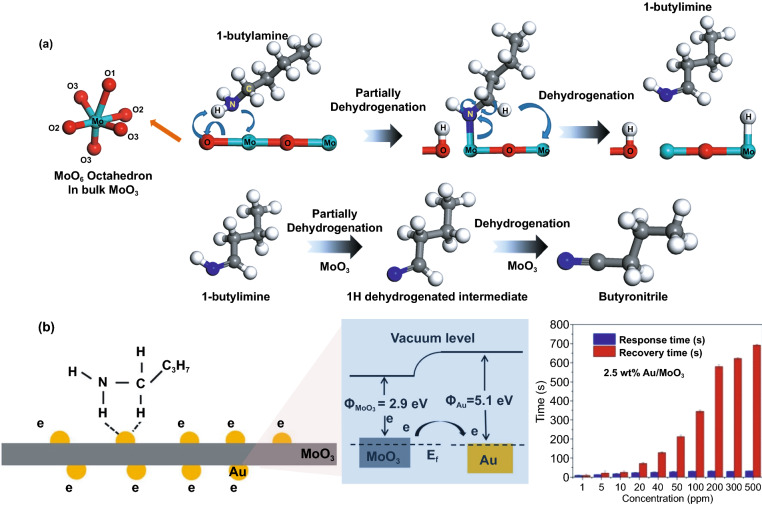
**a** Schematic diagram of the sensing mechanism toward 1-butylamine on the MoO_3_ surface. The dehydrogenation pathway and the electron transfer to the Mo site are displayed. The liberated proton over Mo and the adsorption OH are assumed to adsorb as water. **b** Proposed mechanism of the adsorbed dehydrogenation process on Au/MoO_3_ nanocomposites and its consequence to response and recovery time. Adapted with permission from Ref. [106] Copyright 2021 Elsevier B.V

Decoration of Au on MoO_3_ successfully improved toluene [96], xylene [96], ethanol [45], H_2_S [26, 111], and 1-butylamine [106] sensing performances. As toluene and xylene sensors, the α-MoO_3_ hollow spheres with 450 nm in diameter have been prepared by the solvothermal method, followed by chemical reduction of Au at 120 °C. The Au nanoparticles have a diameter in the range of 10–25 nm. The higher numbers of chemisorbed oxygens on Au decorated α-MoO_3_ hollow sphere than in its pure one increase response 4.6 and 3.9 times at 250 °C to 100 ppm of toluene and xylene, respectively, higher than the pure one at 290 °C [96]. Aside from operating temperature, the presence of a certain amount of Au nanoparticles also reduces response times from 19 and 6 s to 1.6 and 2 s for toluene and xylene, respectively. Moreover, Au preference to coordinate with the aromatic ring group may improve Au–MoO_3_ selectivity to toluene and xylene. The Amount and distribution of Au or noble metal also affect the sensing performance. A high amount and good dispersion of noble metal nanoparticles on the surface of metal oxide raise the catalytic effect yet cover the active sites of oxide leading to decreased performance [112]. A low amount of the metal is distributed sparsely, causing a lower catalytic activity and synergetic effect. Therefore, the proper ratio of metal/metal oxide is vital for achieving the best sensing performance. In the previous case, optimal amount of Au to deliver the highest performance was 2.04% of α-MoO_3_. A different shape leads to a different sensor preference. 4 wt% of Au decorated MoO_3_ nanosheet was reported to sense 200 ppm of ethanol better at its optimum temperature of 280 °C with the response and recovery times of 14 and 5 s, respectively [45]. The nanosheet with the size of 600 nm was prepared using a solvothermal method, while 10–15 nm of Au decoration was performed using the chemical reduction technique. However, the pure MoO_3_ has a lower operating temperature, though the response value is much lower than the decorated one. The same amount of Au was used to decorate 200 nm in a width of MoO_3_ nanobelt and was reported selectively in response to the presence of 1-butylamine [106]. The material preparations were similar to the previous work [45]. Compared to the pure MoO_3_ nanobelt with an optimal operating temperature of 340 °C, the Au decorated nanobelt shows the best performance at 240 °C with a response value of ~ 300. The high selectivity to 1-butylamine is caused by a nitrogen atom in 1-butylamine that has electrons lone pair and bind with acid-Lewis site of Mo ions (Fig. 5a). Moreover, the hydrogen atoms in the gas also support the secondary dehydrogenation producing more electrons. However, strong interaction between the Au decorated MoO_3_ and the gas result in a long recovery. Hence the response time was much shorter than recovery time in both pure and decorated cases, as seen in Fig. 5b [97, 113, 114].

A lower operating temperature is achieved after an additional of Ag to α-MoO_3_ nanorods. The nanorods possess the length and diameter of 10 µm and 200–300 nm, respectively. The Ag nanoparticles on the nanorod surface has a size of ~ 20 nm. The structure was obtained by the solvothermal method to produce nanorods, then Ag reducing by wet chemical reduction at 50 °C. Also, 100 ppm of TEA was detected with the response value of 400.8 at 200 °C with high selectivity [99]. This value is three times greater than its pure counterpart and detects the gas within 3 s. Similar to the previous case, the strong interaction between amine-contained gas and Mo ions leads to an incomplete recovery. To solve the problem, He et al. [101] proposed short-time pulse heating at high temperatures and established that pulse heating at 300 °C for 1 min completes the recovery in 107 s. At the same temperature, Pd-loaded MoO_3_ flower-like nanobelts detect NO_2_ gas with good selectivity [101]. The nanobelt was prepared using chemical spray pyrolysis (CSP) on a glass substrate with MoCl_5_ as a Mo source. The Pd loading was done by dipping the MoO_3_ film in PdCl solution several times and heat at 200 °C to remove the chlorine compound. The pure nanobelts achieves a response of 68% to 100 ppm of NO_2_ at 200 °C. After Pd's addition, the response value increased to 95.3%, with response and recovery time of 74 and 297 s, respectively. The higher affinity of NO_2_ causes the high selectivity to NO_2_ compared to pre-adsorbed oxygen and other gases; hence the NO_2_ chemisorption is preferentially on the Pd-loaded MoO_3_ surface. In another case, the addition of Pt nanoparticles on the α-MoO_3_ nanobelts effectively detected formaldehyde at room temperature [100]. The nanobelts with 200–400 nm in width were prepared using a hydrothermal method, while Pt decoration was performed using the chemical reduction technique. A proper amount of Pt on the nanobelts had a response of 39.3% to 200 ppm of formaldehyde with a response and recovery times of 21.4 and 16.6 s, at room temperature. The presence of Pt nanoparticles raises the response by almost six times of the bare α-MoO_3_.

Functionalization of MoO_3_ with noble metal has been proven to increase response and decrease response time. Although the optimal temperature of MoO_3_ is lower with noble metal functionalization, the reported optimal temperature is still relatively high, which is in the range of 200–250 °C. The combination of ultrathin 2D MoO_3_ and noble metal has the potential to be a superior gas sensor at low temperatures. In addition, this strategy has not been able to overcome the negative effect of humidity. In general, the best performance of gas sensors based on noble metal functionalized MoO_3_ is obtained with a humidity of less than 40% and significantly decreases with increasing humidity [26, 99, 100, 106, 115]. Therefore, further exploration to overcome these challenges needs to be carried out in the future.

### Elemental Doping

Aside from noble metals, other metals are also useful in improving MoO_3_ sensing performance. For instance, small quantities of iron (Fe), nickel (Ni), zinc (Zn), and chrome (Cr) raised the sensor response significantly. The possible reasons for the sensing improvement are believed to be as follows. First, metals increase the porosity of the metal oxides. Second, the high oxidation state of Mo allowed many lower-state metals to replace the Mo site and create an acceptor level. This substitution increases the resistance hence modulating sensing performances. Third, charge balancing compensation generates the oxygen vacancies that increase oxygen chemisorbed species on the oxide surface [116–118]. The metal-doped MoO_3_ preparation and its sensing performance are discussed efficiently in this section.

**Fig. 6:**
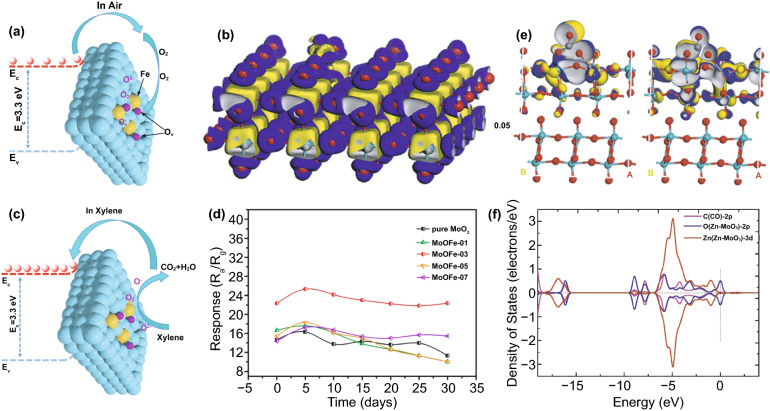
Schematic diagram of **a** Fe-doped MoO_3_ sensor and **b** its electronic density difference isosurfaces when exposed to oxygen molecules. **c** Schematic diagram and **d** long-term stability of Fe-doped MoO_3_ sensor when exposed to xylene. (**a, c, d**) are reprinted with permission from Ref. [120]. Copyright 2020, Elsevier B.V. **b** is reprinted from Ref. [121]. Copyright 2020, Elsevier B.V. **e** HOMO (right) and LUMO (left) and **f** PDOS of CO-adsorbed Zn–MoO_3_ (010) adsorption system. Reproduced from Ref. [122] with permission. Copyright 2020, Elsevier B.V

Fe-doped MoO_3_ with nanobelts and nanoarrays morphologies were prepared by Ruan and Cao groups, respectively [119, 120]. The works were motivated partly by the similarity of the ionic radius of Fe^3+^, 0.064 nm, and Mo^6+^, 0.069 nm, which allow substitution with a minimal defect in the oxide crystal structure. The MoO_3_ nanobelts were prepared using a hydrothermal method with ammonium molybdate tetrahydrate as a Mo source in the water medium [119]. Fe doping was completed by mixing the Mo source with iron nitrate nonahydrate during solution preparation. The pure nanobelts have a width and length of 350 nm and 8 µm, respectively. Interestingly, higher Fe contents increase the tendency of MoO_3_ to form nanosheets structure. However, with the variation of Fe content in the range of 1–15 wt%, 5 wt.% became the optimal amount in detecting xylene. The optimal temperature for xylene detection is 206 °C, with a response value of 6.1. The response and recovery times were recorded as 20 and 75 s, respectively. Moreover, the pure nanobelts show a response value of 2.9 at the same temperature to 100 ppm xylene. In another case, Cao’s group prepared MoO_3_ nanoarrays using the solid-state chemical reaction method with a similar Mo source as Ruan’s experiment [120]. The Mo and Fe sources were mixed mechanically with an agate mortar in the presence of PEG-400 and oxalic acid. The mixture was heated at 60 °C for 24 h and calcined at 450 °C for 1 h, resulting in nanoplate arrays of MoO_3_. The Fe^3+^ doping made the plate thinner due to the lattice distortion that hinders crystal growth. In this case, the Fe amounts are ranged between 0.1 and 0.7 wt%, and 0.3 wt% was the optimal amount for the best structure and sensing performance. The optimal temperature for MoO_3_ nanoplates array was 370 °C, 30 °C higher than Fe-doped MoO_3_. Although it works at high temperatures than those in nanobelts case, it shows a response to 100 ppm of xylene of 28.1 with the response and recovery times of 2 and 21–33 s, respectively. The excellent performances of Fe-doped MoO_3_ are caused by the more oxygen vacancies available to facilitate more chemisorption as shown in Fig. 6a. The fact is strengthened by the density functional theory (DFT) calculation conducted by Lei et al*.* [121] The result shows that monolayer MoO_3_ is insensitive toward oxygen molecule and Fe doping increase its molecule adsorption capability. The isosurface analysis (Fig. 6b) found that the oxygen was chemisorbed by capturing 0.2 e from one Fe-doped MoO_3_. Upon exposure to xylene, oxygen molecules interact with it and consequently released the captured electrons to Fe-MoO_3_. Moreover, the sensing material exhibited a stable response value up to 30 testing days (see Fig. 6c, d).

Ni-doped MoO_3_ detects 100 ppm of xylene at the optimal temperature of 250 °C [116]. In Jiang et al*.* [116] synthesis procedure, the 800 nm in diameter of  nanosheet-assembled MoO_3_ spheres were obtained by the solvothermal method. With a similar procedure, adding a small amount of Ni results in the smaller pompon-shaped sphere. The smaller size indicated the role of Ni as a crystal growth inhibitor. Ni^2+^ diameter (0.072 nm), which is higher than Mo^6+^, causes distortion that inhibits the crystal growth. The smaller Ni-doped MoO_3_ size improves the response value to 62.61, 18 times higher than the MoO_3_ nanosphere with good selectivity. The pompons structure detects xylene in only 1 s. The high response is attributed to the more Schottky contact by the small pompons, increasing the resistance. According to Ruan's group, adding 5 wt% Zr to the MoO_3_ matrix changed nanobelts to nanosphere structure [123]. In the absence of Zr, α-MoO_3_, which was synthesized using the solvothermal method at 180 °C for 36 h, has a nanobelt morphology with a length and width of 6 µm and 200 nm, respectively. A similar method was used with the addition of 5 wt% Zr to the Mo solution during synthesis. The presence of α-MoO_3_ spheres assembled by nanobelts with a size of 600 nm was observed, indicating the role of Zr as a morphology modifier. As a xylene sensor, the presence of Zr increases the response of α-MoO_3_ to 100 ppm xylene by three times at 206 °C. Furthermore, α-MoO_3_ shows excellent selectivity to xylene compared to benzene and toluene due to two methyl groups in xylene. This makes it more reactive to Zr, which has good catalytic activity.

Several studies have reported the improvement of sensor performance of α-MoO_3_ to amine compounds, such as triethylamine (TEA) and trimethylamine (TMA) by involving chrome (Cr) [124], cerium (Ce) [125], and tungsten (W) [118] as metal doping. Li et al. [124] reported the fabrication of the nanorods structure of Cr-doped MoO_3_ by mixing MoO_3_ powder produced through solvothermal followed by annealing processes. Doping Cr inhibits the growth of α-MoO_3_ grains, resulting in shorter nanorod than that of pure MoO_3_. A response value of 150.25 was achieved at 200 °C to 100 ppm TEA with a response and recovery of 7 and 80 s, respectively. The relatively short recovery is supported by pulse heating at 300 °C. In TMA detection, Li et al*.* synthesized α-MoO_3_ nanobelts doped with Ce and W [118, 125]. Ce-doped α-MoO_3_ and W-doped α-MoO_3_ were obtained through a solvothermal process in the presence of cerium nitrate and Na_2_WO_4_ as sources of Ce and W, respectively. Ce and W doping result in different optimal temperatures of 240 and 280 °C, respectively. *α*-MoO_3_ nanobelts show a response of 4.7 to 50 ppm TMA at the optimal temperature of 280 °C, while Ce-doped α-MoO_3_ and W-doped *α*-MoO_3_ show a response of 17.4 and 13.8 at their optimal temperature, respectively. Ce and W substitution at the Mo lattice site increases oxygen vacancies, improving the TMA sensor performance. Furthermore, the relatively short recovery times of 20 and 11 s for Ce and W doping, respectively, show superior amine compound detection performance.

The reducing gases, such as CO and H_2_S, are also reported could be detected by modifying MoO_3_ with metal doping. Bai et al*.* [126] examined Cd-doped α-MoO_3_ as an H_2_S sensing material. Cd-doped α-MoO_3_ nanobelts with a 200–800 nm width and a length of several micrometers were synthesized using a simple solvothermal method at 120 °C for 24 h. Analysis using photoluminescence (PL), XRD, and Raman spectroscopy showed Mo^6 +^ substitution with Cd^2+^ generates defects and oxygen vacancies. Furthermore, Cd also narrowed the bandgap of α-MoO_3_, which was strengthened by the DFT study. These phenomena are the reason for the three times increase in the response of Cd-doped α-MoO_3_ to 100 ppm H_2_S at 140 °C (378.5), where pure α-MoO_3_ performs optimally at 170 °C (123.4). In the case of CO detection, *α*-MoO_3_ is modified by metal Zn. Zn-doped α-MoO_3_ was prepared using a solvothermal method with a pH adjustment of 2. Wang et al*.* [122] reported the formation of a hierarchical micro flower α-MoO_3_ with a size of about 2 µm that is composed of nanosheets. The presence of Zn in the α-MoO_3_ lattice inhibits grain growth, leading to thinner individual nanosheets. Based on DFT calculations as displayed in Fig. 6e, f, the interaction between CO and α-MoO_3_ is classified as a weak interaction. In Zn presence, chemisorption of CO on the oxide surface occurs with a charge transfer of 0.451e. Additionally, the narrowing bandgap from 1.447 to 1.167 eV after Zn addition leads to an increase in the conductance of α-MoO_3_. This narrowing is believed to increase the *α*-MoO_3_ response four times at a temperature of 240 °C, where pure *α*-MoO_3_ has an optimal temperature of 260 °C. In another report, Zn metal was also used to dope *α*-MoO_3_ and work as an ethanol sensor at 240 °C [127]. The response of 321–1000 ppm of ethanol was observed during the measurement. However, this value is 15 times higher than that of pure *α*-MoO_3._

Based on the above discussion, metal doping generally has a function as a modifier of the morphology of MoO_3_. The improved performance of the sensors appears to be due to an increase in the number of oxygen vacancies available on the oxide surface. Although the resulting response is relatively high, the challenge of lowering the working temperature of MoO_3_ does not seem to be solved by this strategy because the MoO_3_ only participates in the sensing mechanism. However, Cd-doped MoO_3_ synthesized by Bai et al*.* showed superior performance in detecting H_2_S at a relatively low temperature of 140 °C [126]. This proves that there is an excellent opportunity for further exploration of this strategy. In addition, elemental doping can also be realized with nonmetal doping such as nitrogen, sulfur, selenium, and carbon [128–132]. Nonmetal doping has been reported to alter the electronic structure, reduce the bandgap, increase the amount of oxygen vacancy, increase the gas adsorption capacity, and induce bipolar electrical transport [128, 133]. Although nonmetal doping on MoO_3_ has been relatively widely reported, its exploitation as a gas sensor is still rarely found. This is another challenge in the field of gas sensors, and exploration in the development of nonmetal doping MoO_3_ is still very wide open.

### Heterostructures Coupling

**Fig. 7:**
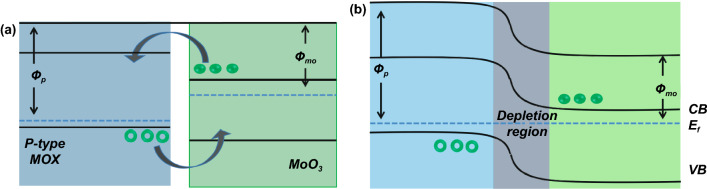
Illustration of band diagram of p-type metal oxide and n-type MoO_3_ **a** before and **b** after making contact. The depletion region is created at the interface as a result of electron–hole recombination during Fermi level alignment

Another strategy to modulate the performance of MoO_3_-based gas sensors is interface modification or heterostructure formation. The modification involves adding other materials, such as other metal oxides, carbon nanomaterials, and polymers. This composite strategy leverages the synergy of two different material properties to achieve superior performance [134, 135]. Response, selectivity, and sensitivity improvement are achieved using this strategy [136–138]. There is a need to consider the ratio of the two materials and the distribution of interface in this strategy because it relates to the conduction path in the surface reaction. Adding p-type semiconductor to n-type MoO_3_ may increase the MoO_3_ resistance due to the depletion region that reduces the MoO_3_ charge conduction channel. The depletion region is created when the p-type semiconductor with a higher work function making contact with MoO_3_. As illustrated in Fig. 7a, the electrons in the MoO_3_ conduction band flow to the p-type conduction band and recombine with holes that flow in the opposite direction. This electron–hole recombination occurs until the Fermi level alignment meets the equilibrium state as shown in Fig. 7b. The depletion region is the region at the interface of the two materials where the major charge carriers of both materials are depleted. This region is believed to be sensitive to the presence of gases. However, the gas sensor performance only can be maximized when the surface reaction is dominated by the depletion region and the MoO_3_ itself. When the number of p-type material is higher or covers the surface of MoO_3_ like in the core–shell case, the conduction path may be fully taken by the p-type materials, and the MoO_3_ does not contribute to the sensing mechanism leading to a lower response. This is why the composition ratio of p-type: n-type is an important key for achieving the best sensing performances. Li et al*.* found that dispersing 50 mg of MoO_3_ nanobelts in ethanol containing 50 mM of Co(NO_3_)_2_.6H_2_O under ultrasonication resulted in CoMoO_4_ decorated MoO_3_ after calcining the product at 500 °C [138]. In this case, CoMoO_4_ acts as a p-type metal oxide that has a narrower bandgap compare to MoO_3_. Moreover, from gas sensor measurement, one can easily find that the fivefold increase in TMA sensing response of CoMoO_4_/MoO_3_ composite at 220 °C is contributed by the p–n junction at CoMoO_4_/MoO_3_. The MoO_3_ itself shows its highest response to 10 ppm of TMA at 280 °C.

In another report, Xu et al*.* [80] examined the p-type of NiCo_2_O_4_ nanosheet coated α-MoO_3_ nanorods. The nanorods themselves were produced using the hydrothermal method with Mo powder as a precursor, while the composite of NiCo_2_O_4_/*α*-MoO_3_ was prepared using a chemical deposition approach. In their typical process, nickel and cobalt nitrates were dispersed in the aqueous solution containing α-MoO_3_ nanobelts powder. The mixture was then heated at 95 °C for 2 h. The composite was obtained after calcining the product at 350 °C for 2 h. These procedures produce nanorods structure with width and length of 200 nm and 20 µm, respectively. Furthermore, the rods were also covered by the NiCo_2_O_4_ nanosheets. In its application as an ethanol sensor, the p–n junction was created at the interface of NiCo_2_O_4_/α-MoO_3_. In general, Fermi alignment occurred along with the electron transfer from the n-type α-MoO_3_ to p-type NiCo_2_O_4_. Since the work function of NiCo_2_O_4_ is lower than that of α-MoO_3_, electron transfer occurred from NiCo_2_O_4_ to α-MoO_3_, leading to a thicker hole accumulation layer on the NiCo_2_O_4_ side. The increase in resistance in the presence of ethanol indicates that the composite follows the NiCo_2_O_4_ characteristic. Under a reducing gas atmosphere, the released electrons from oxygen ion and ethanol reaction resulting in the thinner accumulation layer, leading to increase the composite resistance. This phenomenon is responsible for the high response of composites of 20–1 ppm of ethanol at 350 °C. Furthermore, the acid–base combination in the composites was claimed to have a high selectivity to ethanol.

Aside from p–n junction, n–n junction also can be created by contacting MoO_3_ with another n-type metal oxide. For example, the formation of the n–n junction was realized by decorating MoO_3_ nanobelts with Fe_2_O_3_ nanoparticles [139]. The decoration was completed using hydrothermal in the presence of FeCl_3_·6H_2_O and MoO_3_ nanobelts. The 40 nm of Fe_2_O_3_ nanoparticles on the nanobelts create the n–n junction at its interface. As reported, different work functions between the two materials cause a depletion layer associated with barrier potential. The potential does not only produce the excellent response of 22.48 at 233.5 °C to 100 ppm of xylene but also improves the selectivity to xylene compared to the other VOC gases. Zhang et al*.* [140] prepared the MoO_3_/Bi_2_Mo_3_O_12_ hollow sphere composite via hydrothermal method. Based on the XPS spectra, the conduction band of MoO_3_ is located lower than Bi_2_Mo_3_O_12_; hence the electrons are transferred from Bi_2_Mo_3_O_12_ to MoO_3_. The electron transfer generates the depletion layer at the interface of MoO_3_/Bi_2_Mo_3_O_12_ and its thickness is sensitive to the change of atmosphere. The creation of depletion layer created and the number of oxygen ions trapped at the interface modulate the composite response to 50 ppm of TMA at 170 °C, 2.5 and 5.5 times higher than those of MoO_3_ and Bi_2_Mo_3_O_12_, respectively.

**Fig. 8:**
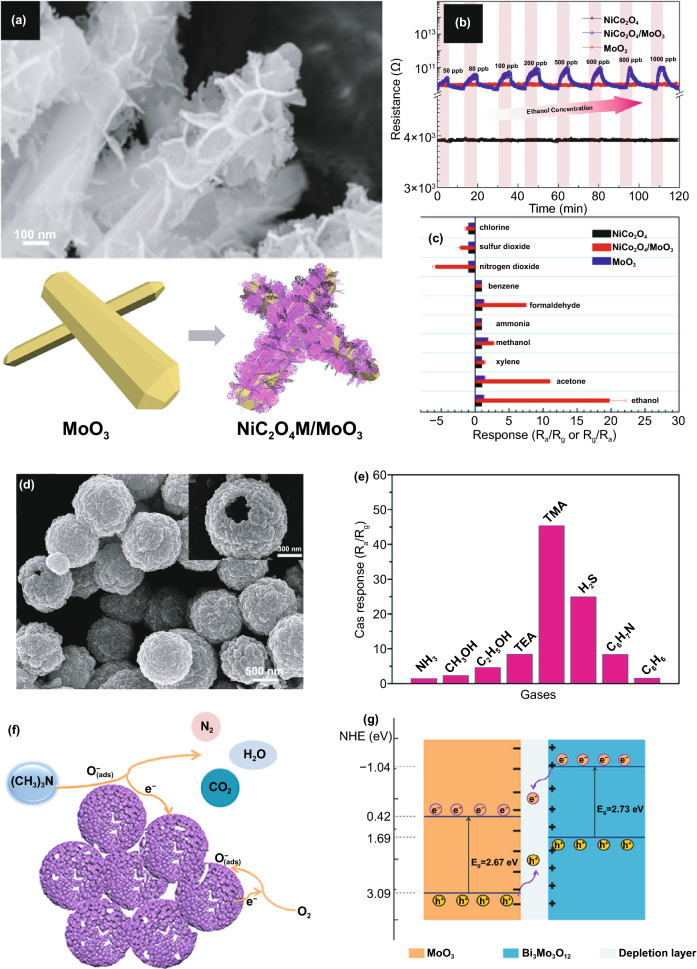
**a** p–n heterostructure was constructed by NiCo_2_O_4_/*α*-MoO_3_ nanorods with (**b, c**) sensing response and selectivity to 1 ppm of ethanol at 350 °C. Reprinted with permission from Ref. [80]. Copyright 2019 Elsevier 2020. **d** Hollow heterostructures consisted of MoO_3_/Bi_2_Mo_3_O_12_ exhibited selective sensing toward TMA **e**. The proposed sensing mechanism (**f, g**) showed electron–hole transfer processes between MoO_3_ and Bi_2_Mo_3_O_12_ upon the contact. Reproduced from Ref. [140] with permission. Copyright 2019 American Chemical Society

Heterostructure coupling can also be formed by combining MoO_3_ with carbon nanomaterials, such as reduced graphene oxide (rGO). The rGO is categorized as 2D materials with remarkable properties and high surface area. Incorporating rGO to α-MoO_3_ provides a conduction channel that increases surface reaction rate and reduces the operating temperature. Bai et al*.* [141] successfully incorporated MoO_3_ nanorod onto the rGO surface with a very good distribution for optimal contact with rGO to be achieved. Sensing examination to 40 ppm H_2_S shows that without rGO, α-MoO_3_ works well at 170 °C with a response of 23.4. With 5 wt% of rGO, the composite works best 110 °C with a response of 59.7. Moreover, the observed response time and recovery time were 9 and 17 s, respectively. With optimal contact between the oxide and rGO, the rGO facilitates gas diffusion channels, mass transport, improve charge separation, and conduction channels between H_2_S and α-MoO_3_. By increasing α-MoO_3_ resistance after rGO incorporation, rGO increases the characteristics of the n-type MoO_3_ [141, 142]. The incorporation of α-MoO_3_ nanoparticles on the surface of rGO for the H_2_S sensor has also been reported [143]. The increasing specific surface area from 770 to 894 m^2^ g^−1^ was achieved after 3 wt% of incorporating the nanoparticles. The enhancement of surface area causes the high response of 4120–100 ppm of H_2_S at 160 °C. Although the operating temperature of α-MoO_3_ nanoparticles-rGO is higher than the nanorod one, they show a better response. Figure 8 represents the work on heterostructures sensor based on *α*-MoO_3_.

As stated earlier, the heterostructure coupling can be realized by combining MoO_3_ with other metal oxide or carbon nanomaterials. Up to now, one can conclude that with proper ratio, high response and relatively low operating temperature can be achieved by p–n or n–n heterojunction. This strategy can also improve the selectivity of the composite. The higher resistance of the composite due to depletion layer formation is more suitable for detecting reducing gas such as TMA and H_2_S. The higher resistance provides a wider detection range and a lower the limit of detection. Furthermore, the high conductivity of carbon nanomaterials at low temperatures is also a benefit for MoO_3_. The highly p-doped carbon nanomaterials also contribute to sensing materials by generating Schottky contact with MoO_3_, therefore, the high response at 100 °C can be achieved. Furthermore, it is expected that hydrophobic of carbon nanomaterials can effectively help MoO_3_ prevent the negative effect of humidity. As reported, carbon nanomaterials such as carbon nanotubes (CNT) and graphene show negligible humidity interfering effect up to 80% at low temperature [144]. However, the study of the effect of carbon nanomaterials on humidity interference in MoO_3_-based gas sensors cannot be found anywhere. Therefore, the further investigation on this problem needs to be carried out in the future.

In summary, morphology design is most effective in increasing response. This strategy is strongly related to the number of active sites that depend on the morphology and specific surface area. The metal catalyst, such as noble metal and elemental doping, reduces the optimal temperature with the increased response. Moreover, the heterostructure strategy is also essential in achieving gas sensors with a high sensitivity, low operating temperature, and low response and recovery times. The parameters, such as the ratio of the second phase and MoO_3_ need to be considered carefully to obtain the best performance. Table 1 summarizes the comparison of all strategies in gas detection.

**Table 1:** Comparison of modification of MoO_3_ technique in various gas detection

Improvement strategies	Sensing materials	Target gas	Conc. (ppm)	T (°C)	Response (*R*_*a*_/*R*_*g*_*)* or (R_air_-R_gas_)/R_air_)	Response/recovery times (s)	Refs.
Morphology design	*α*-MoO_3_ nanowires	H_2_	15,000	RT	0.85%	3/2.7	[48]
	*α*-MoO_3_ nanorods	Ethanol	400	350	35	N/A	[68]
	*α*-MoO_3_ nanobelts	Xylene	100	206	3	7/87	[69]
	*α*-MoO_3_ nanobelts	Ethanol	800	300	174	~ 40/ ~ 5	[70]
	*α*-MoO_3_ nanosheets	Alcohol	100	300	33.1	21/10	[74]
	*α*-MoO_3_ nanoflakes	Alcohol	100	300	28.1	23/13	[74]
	*α*-MoO_3_ sheets	NO_2_	10	250	56%	N/A	[75]
	*α*-MoO_3_ sheets	H_2_S	10	250	18%	N/A	[75]
	Flower-like α-MoO_3_	TEA	10	170	931.2	25/–	[87]
	*α*-MoO_3_ nanobelts	TEA	10	170	114.9	29/–	[87]
	*α*-MoO_3_ nanoparticles	TEA	10	170	27.6	28/–	[87]
	*α*-MoO_3_ nanobelts	Ethanol	200	300	21	69/174	[89]
	*α*-MoO_3_ nanofibers	Ethanol	200	275	53	45/138	[89]
	*α*-MoO_3_ nanorods	Ethanol	100	250	8.9	20/15	[91]
	Sponges-like α-MoO_3_	Ethanol	100	250	19.8	15/15	[91]
	*α*-MoO_3_ microboxes	Ethanol	100	260	78	15/5	[90]
	Sphere-like nanoflowers α-MoO_3_	Ethanol	300	300	30.9	N/A	[85]
	Rose-like nanoflowers α-MoO_3_	Ethanol	300	300	37.1	N/A	[85]
	Plate flowers α-MoO_3_	Ethanol	300	300	27.3	N/A	[85]
	Nanosheet-assembled hierarchical MoO_3_	Ethanol	400	300	32	13/9.6	[92]
	Nanofiber-assembled hierarchical MoO_3_	Ethanol	400	300	24	3.2/2.4	[92]
Surface functionalization with noble metals	Au decorated α-MoO_3_ hollow sphere	Toluene	100	250	17.5	1.6/-	[96]
	Au decorated α-MoO_3_ hollow sphere	Xylene	100	250	22.1	2/-	[96]
	Au decorated MoO_3_ nanosheet	Ethanol	200	280	169	14/5	[45]
	Au decorated MoO_3_ nanobelts	1-butylamine	100	240	~ 300	23/388	[106]
	Ag decorated α-MoO_3_ nanorods	TEA	100	200	400.8	3/107	[99]
	Pd-loaded MoO_3_ flower-like nanobelts	NO_2_	100	200	95.3	74/297	[101]
	Pt loaded α-MoO_3_ nanobelts	Formaldehyde	200	RT	39.3	21.4/16.6	[100]
Elemental doping	Fe-doped MoO_3_ nanobelts	Xylene	100	206	6.1	20/75	[119]
	Fe-doped MoO_3_ nanoarrays	Xylene	100	340	28.1	2/21–33	[120]
	Ni-doped MoO_3_ pompons	Xylene	100	250	62.6	1/50	[116]
	Zr-doped *α*-MoO_3_ nanobelts	Xylene	100	206	7.99	32/264	[123]
	Cr-doped MoO_3_ nanorods	TEA	100	200	150.25	7/80	[124]
	Ce-doped *α*-MoO_3_ nanobelts	TMA	50	240	17.4	10/20	[125]
	W-doped *α*-MoO_3_ nanobelts	TMA	50	280	13.8	6/11	[118]
	Cd-doped *α*-MoO_3_ nanobelts	H_2_S	100	140	378.5	23/45	[126]
	Zn-doped *α*-MoO_3_ microflower	CO	50	240	31.23	10/14	[122]
	Zn-doped *α*-MoO_3_ nanobelts	Ethanol	1000	240	321	N/A	[127]
Heterostructure	NiCo_2_O_4_ nanosheet coated *α*-MoO_3_ nanorods	Ethanol	1	350	20	N/A	[80]
	Fe_2_O_3_–MoO_3_ nanobelts	Xylene	100	233.5	22.48	4/102	[139]
	MoO_3_/Bi_2_Mo_3_O_12_ hollow sphere	TMA	50	170	25.8	7.1/–	[140]
	rGO–MoO_3_ nanorod	H_2_S	40	110	59.7	9/17	[141]
	rGO–*α*-MoO_3_ nanoparticles	H_2_S	100	160	4120	–/120	[143]

## Molybdenum Disulfide (MoS_2_) Gas Sensing Materials

**Fig. 9:**
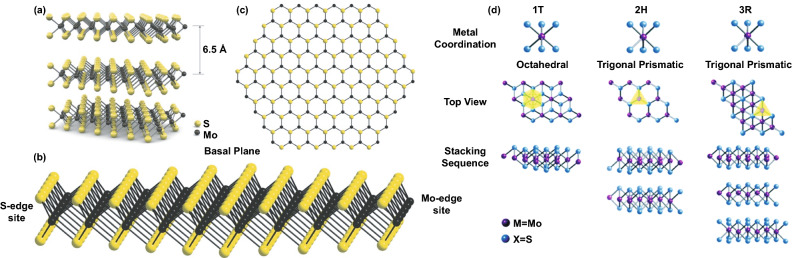
**a** 3D form of bulk MoS_2_ and **b, c** side and top views of 2D hexagonal layouts of single-layer MoS_2_ (H‐MoS_2_). Reprinted with permission from Ref. [154]. Copyright 2011, Nature Publishing group. **d** Metal coordination and stacking sequences of TMD structural unit cells. Metal coordination can be either octahedral or trigonal prismatic. The octahedral coordination allows stacking sequences, which yield a tetragonal symmetry (1 T). Dissimilar stacking sequences of trigonal prismatic single layers give rise to different symmetries, including hexagonal symmetry (2H) and rhombohedral symmetry (3R). Reproduced with permission from Ref. [148]. Copyright 2017, The Royal Society of Chemistry

Molybdenum sulfide (MoS_2_) is naturally available as a bulk molybdenite crystal with a 2H phase as a thermodynamically stable form. It exhibits an indirect band gap property of approximately 1.2 eV [145]. According to Fig. 9, the bulk possesses an interlayer space of 0.65 nm allows further delamination. It can be transformed into MoS_2_ single-layer structures with a large intrinsic bandgap of 1.8 eV by mechanical exfoliation [146]. MoS_2_, in the bulk form, has different crystal phases depending on the coordination bonding and stacking orders of [MoS_6_] polyhedral. In general, MoS_2_ crystallizes in three phases, including hexagonal (2H), octahedral (1 T), and rhombohedral (3R) with identical vertically stacking layers [147, 148]. There are strong in‐plane covalent bonds of two sulfur atoms-sandwiched molybdenum atoms bounded by weak van der Waals forces [149]. Although they have similarities in their structures, only hexagonal 2H-MoS_2_ with trigonal prismatic coordination behaves like a metal. 1 T-octahedral coordination (1T-MoS_2_) and rhombohedral structure 3R-MoS_2_ with trigonal prismatic coordination exhibit metals or semimetals characteristics [150]. There are five polymorphs in the single crystal or monolayer structure of MoS_2_, including 1H, 1 T, 1 T′, 1 T′′ and 1 T′′′ [151]. Trigonal prismatic and octahedral coordination of bulk crystals are inherited by 1H-MoS_2_ and 1 T-MoS_2_, respectively. However, in monolayer phases, some point group symmetry changes lead to different inversion symmetries, such as D_6h_ to D_3h_ in the 1H-MoS_2_ case. 1 T′, 1 T′′, and 1 T′′′ phases form due to the distorted structures of [MoS_6_] octahedra [152, 153].

The significant variation of crystal structures (bulk and monolayer) and phases (stable and metastable) that MoS_2_ possess bequeaths the unique features in their properties, such as tunable optical band gap (1.2–1.8 eV) and electronic structures [155]. Mechanical properties of MoS_2_ are previously investigated. Bertolazzi et al*.* [156] have measured some mechanical characteristics of ultrathin MoS_2,_ which consists of a few layers. The MoS_2_ monolayer exhibited in-plane stiffness of 180 ± 60 N m^–1^, corresponding to an effective Young's modulus of 270 ± 100 GPa higher than its bulk MoS_2_ counterpart (240 GPa) and benchmark carbon steel (210 GPa). Furthermore, the 2D monolayers have high stretchability and flexibility upon applying mechanical force without losing their inherited properties [157]. The monolayer MoS_2_ has a breaking strength of 22 ± 4 GPa, which is about 11% of its Young’s modulus [156]. According to the literature, Bulk MoS_2_ shows electron mobility of 0.5–3 cm^2^ V^−1^ s^−1^ [158]. The mobility can be increased to 12.1 cm^2^ V^−1^ s^−1^ by making the monolayer MoS_2_ into polycrystalline nature [159]. The highest electron mobility (200 cm^2^ V^−1^ s^−1^) was achieved in a single-layer MoS_2_ transistor [154]. The electrical conductance of monolayer MoS_2_ was 1.3 × 10^−5^ Ω cm^−1^ at room temperatures [160], which can be further increased through substitutional atomic doping, such as Nb and Re [161]. Additionally, 1 T-MoS_2_ has seven times higher conductivity than 2H phase and smaller contact resistance for FETs (200–300 Ω μm at zero gate bias for 1 T-MoS_2_ and 0.7–10 kΩ μm for 2H-MoS_2_) [162, 163]. Both bulk and monolayer MoS_2_ also exhibit excellent thermal conductivity. The experimental works showed that the out-of-plane thermal conductivity of bulk MoS_2_ at 300 K falls within 1–52 W m^−1^ K^−1^ range and depends on the layer thickness of MoS_2_ [164–167].

The last parameter that affects the gas sensing properties of MoS_2_ is chemical. In this review, the chemical property is limited to surface chemistry properties since the gas sensing reaction and charge transfer process occurs mainly at the material surface. Therefore, knowledge and understanding of the surface chemistry nature of MoS_2_ are essential in advancing gas sensing properties. Surface-active sites differ in each MoS_2_ phase. 2H-MoS_2_ has highly surface-active for chemical adsorption edges at their layers [168, 169]. In 1 T-MoS_2_, the surface-active is located in both edges and activated basal plane [170]. Therefore, 1 T-MoS_2_ is more promising for chemical adsorption technology, such as catalysts and sensors [171, 172]. In a typical XRD pattern of MoS_2_, three main peaks emerge. The inert basal plane has an orientation of (002) crystal plane, while (100) and (103) planes correspond to a step and edge plane, respectively.

**Fig. 10:**
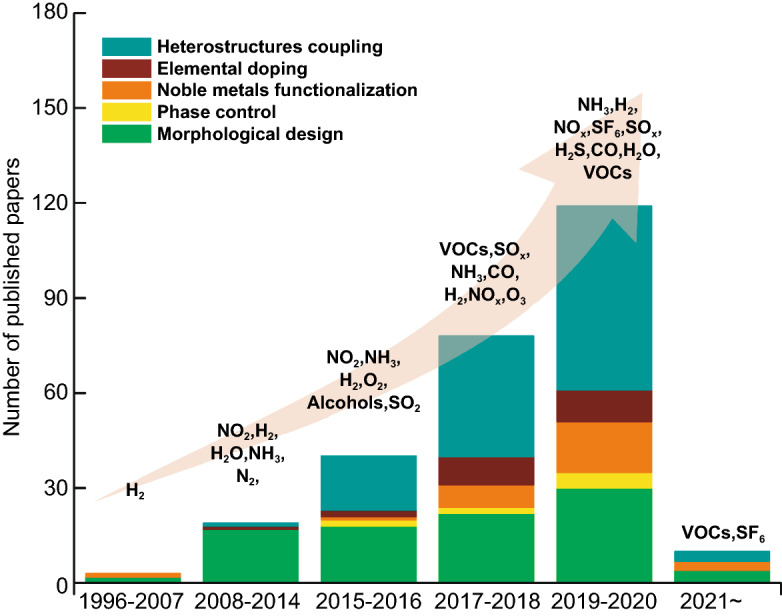
Number of publications reporting advanced strategies to enhance gas sensing properties of MoS_2_ toward various gas detection. Data are obtained from Web of Science (WoS) as of April 2, 2021, with the keyword “MoS_2_ gas sensing.” Both experimental and computational reported are included. Review and perspective articles are excluded

We collected literature of MoS_2_-based sensors available from the WoS database shown in Fig. 10. The first work on the MoS_2_-based gas sensor was published in 1996. Similarly, MoS_2_-based gas sensors were only available in thin-film structures. The sensing investigation of MoS_2_ was limited to non-carbon-containing gases. A great interest in MoS_2_-based sensors began not over a decade ago, where the significant improvement of their gas sensing performance was made by coupling with other materials. Moreover, this approach is still the most popular strategy for MoS_2_ because of its interesting electronic structures that can support the performance of most oxide-based materials. Designing various morphological nanostructured MoS_2_ is more feasible by wet chemical synthesis, although they possess layered structures. Advanced knowledge of phase diversity in MoS_2_ structure expands the new strategy on how 1 T-2H phases engineering affects the gas sensing properties. Different from that of MoO_3_, the MoS_2_ is more sensitive to non-volatile organic compound (VOCs) gas due to the non-catalytic properties of MoS_2_. However, using noble metals-functionalized surface strategy, it is also possible to detect VOCs highly. It should be noted that the majority of MoS_2_-based sensors can be operated at room temperature.

### Insight into Gas Sensing Mechanism of MoS_2_

**Fig. 11:**
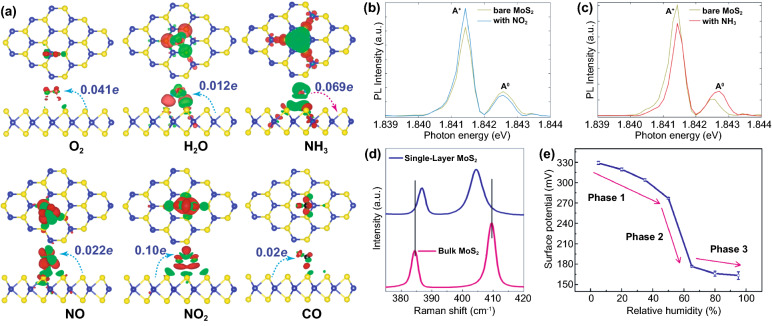
**a** Charge density difference plots for O_2_, H_2_O, NH_3_, NO, NO_2_, and CO interacting with monolayer MoS_2_. The red (green) distribution corresponds to charge accumulation (depletion). The isosurface is taken as 5 × 10^−4^ e Å^−3^. The direction and value of charge transfer are also denoted. Reprinted from Ref. [175]. Copyright 2013, Springer Inc. In situ PL spectra recorded from the MoS_2_ with **b** NO_2_ and **c** NH_3_ molecules. Reprinted from Ref. [174]. Copyright 2015, Springer Nature. **d** Raman spectrum of bilayer MoS_2_ sheet before and after exposure to 1000 ppm NH_3_. Reproduced from Ref. [176] with permission. Copyright 2013, American Chemical Society. **e** Statistical results of the Surface Potential of monolayer MoS_2_ under different relative humidity. Reproduced from Ref. [177] with permission. Copyright 2017, IOP Publishing, Ltd

The gas detection mechanism by MoS_2_ is still debatable. Some researchers believe that the gas sensor mechanism of MoS_2_ is similar to oxide-based materials where the oxygen reduction and oxidation process during gas detection is involved. In contrast, others believe that the gas sensor mechanism of MoS_2_ is a direct charge transfer from or to MoS_2_, which directly affects its conductivity [173, 174]. However, the recent experimental and theoretical evidence have shown straightforward proof that the gas sensor mechanism of MoS_2_ is a charge transfer process. Yue et al*.* [175] have reported the theoretical study of the molecular adsorption process of MoS_2_. Various gases, including the H_2_, O_2_, NH_3_, NO, NO_2_, H_2_O, and CO gases, have been investigated to be adsorbed on the MoS_2_ surface. Figure 11a shows the charge density difference of all gases interacting with the MoS_2_ calculated by Bader charge analysis. It could be seen that the charge transfer process occurred from or to the MoS_2_ surface. Different gases result in different charge transfer behaviors due to the chemical structure of the gases molecules. The H_2_, O_2_, NO, NO_2_, H_2_O, and CO gases received the electron from the MoS_2_ surface, which indicates the electron acceptor behavior of these gases. On the other hand, in NH_3_ gas, the NH_3_ donates the electrons into the MoS_2_ surface. This phenomenon will affect the conductivity of MoS_2_, which will be detected as the change of the electrical signal during the gas detection. For example, in the case of NO_2_, which acts as electron acceptor gas, the conductivity of n-type MoS_2_ will decrease due to the reduction of its charge carrier (electron) number from the n-type MoS_2_ surface. On the other hand, in NH_3_, because it acts as an electron donor gas, the conductivity of n-type MoS_2_ will increase due to the additional electron on the surface [176].

The experimental evidence about the charge transfer process during the gas sensor measurement has also been investigated through several methods. Cho et al. [174] have conducted the in situ photoluminescence (PL) analysis of MoS_2_ in the presence of NO_2_ and NH_3_ to understand the interaction between MoS_2_ and thus gases. Figure 11b, c shows the in situ photoluminescence measurement results of MoS_2_ in the presence of NO_2_ and NH_3_ gases. The A exciton signal from MoS_2_ can be expanded into two species: a trion of A^−/+^ (two electrons to a hole, resulting in a negatively charged exciton, or an electron to two holes, resulting in a positively charged exciton) and a neutral exciton of A^0^. The PL analysis after and before gases exposure is shown in Fig. 11b, c. The A^+^ and A^0^ trion appeared in the PL spectra. After NO_2_ gas exposure, the A^+^ and A^0^ peak intensity change. The A^+^ trion increase after NO_2_ gas exposure while the A^0^ peak intensity decrease. This phenomenon occurred because of the electron deficiency in the MoS_2_ after NO_2_ adsorption. Another report from Kelement et al*.* [180], who studied the Fermi energy of MoS_2_ under N_2_ and O_2_ atmosphere, has also confirmed the charge transfer between MoS_2_ and O_2_ gases through PL measurement. A relative spectral weight shifts from A^−^ to A^0^ during the oxygen exposure, and the PL intensity increases. This behavior occurred due to the depletion of electrons which in this case is of chemical origin. Because O_2_ is more electronegative than N_2_, the ion sorption of O_2_ as O^2−^ results in the depletion of free electrons due to charge transfer to O_2_ molecules. The Raman analysis has been confirmed able to detect the charge transfer process between MoS_2_ and the gases. Figure 11d shows the Raman spectra of the as-prepared MoS_2_ and as-prepared MoS_2_ in the presence of NH_3_ investigated by Late et al*.* [178]. The Raman A_1g_ and E_2g_ peaks' shifting was observed, which attributed to the charge transfer interaction with an electron donor molecule [181, 182]. Feng et al*.* [183] have conducted the potential surface analysis under different humid air environments by using Kelvin probe force microscopy. The result plotted in Fig. 11e has shown that the surface potential of the MoS_2_ decreases with the increase in humidity value. The decrease in the surface potential is due to the injection of carriers from the adsorbed water led to the Fermi level shift of MoS_2_. From all this analysis, it is confirmed that the gas detection of MoS_2_ is a charge transfer process.

### Morphological Design

With a lamellar structure, it is quite demanding to design various morphological structures of MoS_2_. Most of the synthesized MoS_2_ exhibited either monolayer, few layers, or multilayer structures. The sensing materials morphology is usually designed to optimize the gas adsorption/desorption processes, such as with more active sites, large surface area, porosity, or surface defect, leading to improved gas sensing properties. With an appropriate approach and synthesis method, the shape of MoS_2_ could be altered into different dimensions. A good example is the use of surfactants in hydrothermally synthesized MoS_2_. A controlled morphology, including spherical, bulk-like, and flower-like MoS_2,_ was produced by varying surfactants, such as PEG, SDS, PVP, AOT, or CTAB [178–180]. Other experiments involving surfactant-assisted hydrothermal process successfully fabricated some shape variants of MoS_2,_ such as 1D nanoribbons [181], 2D nanoplatelets [182], 3D hollow nanoparticles [183], and 3D hierarchical microspheres [184, 185]. The remaining surfactants may become an impurity in the synthesized products, amplifying the functional performance. However, this leads to alternative surfactant-free synthesis for morphology-controlled MoS_2_, which might be of great interest to many researchers. Sen et al*.* [186] and Ye et al*.* [187] fabricated 2D nanowalls and bilayer nanosheets without involving any surfactant or directing agent. In many cases, with or without surfactants, morphological features and shape tunability of MoS_2_ can be successfully performed.

**Fig. 12:**
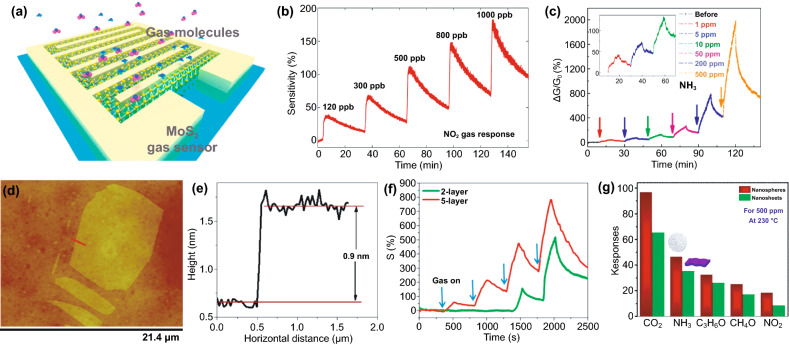
**a** 3D schematic image of the MoS_2_ gas sensor device under gas molecules. **b** The transient NO_2_ gas response of the MoS_2_ device from 120 to 1000 ppb at RT. Adapted from Ref. [193]. Copyright 2015, American Chemical Society. **c** Real-time conductance change in MoS_2_ FETs with time after exposure to NH_3_ under different concentrations, adapted with permission from Ref. [194]. Copyright 2014, American Chemical Society. **d** AFM image and **e** corresponding AFM height profile of single-layer MoS_2_ sheet deposited on 300 nm SiO_2_/Si substrate using the micromechanical cleavage method. **f** Comparative two- and five-layer MoS_2_ cyclic sensing performances with NO_2_ (for 100, 200, 500, 1000 ppm). Reprinted from Ref. [176]. Copyright 2013, American Chemical Society. **g** Selectivity of MoS_2_ nanostructures for different gases, reproduced from Ref. [188]. Copyright 2018 Elsevier

This section discusses how different morphologies influence the gas sensing properties of MoS_2_, including those layered structures and other morphologies. As a native structure, monolayer MoS_2_ is among the primary gas sensing material due to its high surface-to-mass ratio. Other studies show that 2D monolayer structures sense chemical vapors, NO_2_, H_2_, and CO gases [32, 176, 188–192]. Figure 12a, b shows some selected works on gas sensing performances of 2D mono-/single-layer MoS_2_ in NO_2_, trimethylamine, and ammonia detection. Notably, edge sites of 2D MoS_2_ monolayer are more reactive than the basal planes. For this reason, constructing MoS_2_ with a dominant edge site improves the sensitivity to several folds. The first principle study suggested that hydrogen molecules are favorably adsorbed on the top of Mo atoms at the edge site rather than Mo atoms at basal planes that strongly supports the experimental results [189]. The ability to respond to a wide range of low concentration gases, mono-/single-layer MoS_2_ makes it an ideal sensing material. Moreover, such a structure offers greater flexibility with retained properties upon mechanical bending, compressing, and stretching [44]. Whether 2D MoS_2_ monolayer is an optimized structure to obtain high-performance sensing is still under debate. Sensing devices comprising thin-layered MoS_2_ with different thicknesses were fabricated by micromechanical exfoliation mounted on the chip [176]. The thickness of single-layer MoS_2_ is about 0.9 nm, as confirmed by AFM (Fig. 12d, e). The results showed that the five-layer MoS_2_ sample has better sensitivity to NH_3_ and NO_2_. However, the enhanced sensing mechanism is still unclear because MoS_2_ may exhibit different electronic structures and redox mechanisms when the layered structures are altered. This issue limits further understanding of the solid–gas interaction at the interface of single- and multilayer 2D MoS_2_ and overcoming this issue requires special attention. DFT calculation can be a good approach attempting for the revelation of electronic structure dependency in a single- and multilayer 2D MoS_2._

Although 2D mono-/few layers MoS_2_ have outstanding performances in sensitivity, selectivity, low-power consumption and stability, their complex synthesis process, and device fabrication are not favorable for scaling-up production to mass application. However, 3D hierarchical nanostructures assembling from the lower dimension of 2D nanocrystal provide a simpler and scalable synthesis [195]. Particularly, their shorter diffusion pathway, relatively higher surface area, and distinguished electronic properties compared to conventional 2D structures increase the interaction with adsorbed molecules, leading to higher responsivity. For instance, the 3D hierarchical MoS_2_ nanospheres exhibited excellent sensing properties to CO gas at 230 °C, which surpassed the performance of 2D nanosheets, as shown in Fig. 12g. The CO sensing properties were not observed previously in any other 2D MoS_2_ [188]. In similar cases, 3D hierarchical porous MoS_2_ synthesized by a simple hydrothermal method had different gas selective properties, including NO_2_ and H_2_ [185]. It gives novel knowledge on tunable gas selectivity by precise morphological design. However, comprehensive works are needed to understand tunable selective properties on different crystal morphologies.

The gas sensing performance of lower-dimensional MoS_2_ (0D and 1D) is far less investigated, although 0D and 1D MoS_2_ fabrications are feasible, and they are substantial components in several applications, including electrocatalysis and energy storage. 0D MoS_2_ can be prepared by a top-down and bottom-up approach. In the top-down process, 2D MoS_2_ undergoes thinning and bond-braking processes with the aid of ion intercalation, chemical/liquid exfoliation, or sonication. On the other hand, the hydrothermal reaction has been a convenient pathway in producing 0D MoS_2_ by a bottom-up process. The synthesis involves Mo and S precursors in aqueous media. Generally, the size of produced 0D MoS_2_ is in a range of 0.5–4.5 nm. Due to this quantum size confinement, 0D MoS_2_ exhibits abundant active sites, large surface areas, and a large band gap (> 3.96 eV), raising unique gas sensing properties. Nevertheless, using 0D MoS_2_ for gas sensing is challenging because it easily gets agglomerated, reducing its active surface areas. Thus, supporting materials are required to provide the anchor platform. The 1D MoS_2_ (nanowires, nanotubes, nanoribbons, etc.) has also been successfully fabricated in a similar approach. MoS_2_ nanotube, for example, was synthesized by chemical transport using MoS_2_ powder as a precursor and iodine as a transport agent [196]. It had, however, size nonuniformity, defective structure, and low yield. The low-temperature hydrothermal method offers an alternative to synthesize 1D MoS_2_ nanotube and nanorod with high size homogeneity and high yield. Benefiting from the enhanced surface-to-volume ratio and the faster charge transfer along the length direction, high-performance gas sensing can be enabled. It is, therefore, expected that both 0D and 1D MoS_2_ would boost the detection of various gases due to the facts described above. Nevertheless, this hypothesis needs theoretical and experimental validation.

### 1T—2H Phase Control

**Fig. 13:**
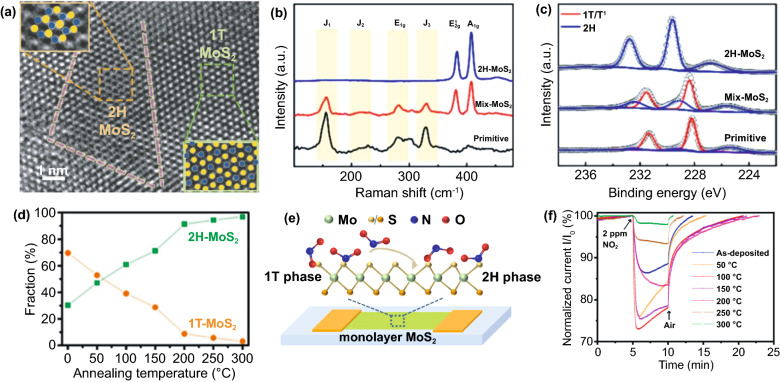
**a** HRTEM images of 1 T/2H MoS_2_**.** Reproduced with permission from Ref. [201]. Copyright 2018, Wiley-VCH. **b** Raman spectra and **c** XPS spectra for the primitive 1 T’-MoS_2_ nanosheets. The mix-MoS_2_ represents the exfoliated MoS_2_ nanosheets obtained through butyl lithium intercalation. Reproduced from Ref. [202] with permission. Copyright 2017 The Royal Society of Chemistry. **d** Relative fractions of 1 T and 2H phases as a function of temperature. **e** Schematic illustration of NO_2_ detection of 1 T/2H MoS_2_. **f** Sensing responses of as-deposited and thermal-annealed ML-MoS_2_ to 2 ppm NO_2_ gas. Reproduced with permission from Ref. [207]. Copyright 2020 American Chemical Society

The recent development of gas sensor devices still focuses on semiconductor-like 2H-MoS_2_. However, the 2H-MoS_2_ has limitations, primarily due to limited active sites and small adsorption energy. Several studies show that the active sites of 2H-MoS_2_ are only located on the edge of the crystal structure, while the abundant basal plane is inert for chemical reactions [197–199]. In comparison, the 1 T/1 T’ of MoS_2_ is more active than 2H-MoS_2_. Tang et al*.* [200] studied the adsorption performance of various molecules, including H, CH_3,_ CF_3_, OCH_3_, and NH_3_. The results showed that the adsorption energy of 1 T and the molecular adsorption ability of 1 T MoS_2_ were significantly higher than 2H-MoS_2_. However, the 1 T-MoS_2_ itself is electrically conductive to be applied as a sensor; hence electrical change during the molecular adsorption was hardly observed. The 1 T/1 T’ phase is relatively unstable, and therefore, it only exists in the mixed phases of 1 T/2H-MoS_2_. The HRTEM image (Fig. 13a) showed the observed grain boundary between orthorhombic and tetragonal structures, which indicates the successful formation of 1 T/2H-MoS_2_ [201]. The electronic properties of 1 T/2H-MoS_2_ are easily understood by Raman and XPS analysis, as shown in Fig. 13b, c [202]. The Raman spectra of 1 T/2H-MoS_2_ consist of several vibration peaks. Three peaks located at 156, 228, and 330 cm^−1^ are attributed to the J_1_, J_2_, and J_3_ vibration modes of the 1 T phase. The vibration peaks located at 283 and 403 cm^−1^ are attributed to the E_1g_ and A_1g_ modes. The formation of 1 T/2H-MoS_2_ can be analyzed by XPS of Mo 3*d* core spectra, as shown in Fig. 13c. The Mo 3*d* core-level spectra of 1 T/2H-MoS_2_ are deconvoluted into four different peaks. The lower binding energy peaks are attributed to the 1 T phase, while the higher binding energy peaks belong to 2H-MoS_2_.

Several reports have demonstrated the formation of 1 T/2H-MoS_2_ for various kinds of applications, such as hydrogen evolution reactions [201, 203, 204], hydrodesulfurization [205], and gas sensor applications [172]. Yang et al*.* [206] demonstrated the formation of the 1 T/2H-mix phase in the molybdenum tungsten sulfate (MWS_2_) system for acetone gas detections. Hydrothermal reactions achieved the mixed 1 T/2H phases. With further annealing, the 1 T phase turned into a 2H phase. The enhanced acetone detection performance to several folds was achieved with only 10% of 1 T content in the MWS_2_ system. Taufik et al*.* [172] have also successfully demonstrated the formation of 1 T/2H-MoS_2_ structure via ethylene glycol (EG) intercalation for improved toluene gas detection performance. The EG intercalation process enhanced the ratio 1 T/2H phase from 1.7 to 4.0 and decreased the conductivity of 1 T/2H-MoS_2_ due to EG low conductivity. The electron from toluene is transferred to the MoS_2_ surface during the toluene adsorption, increasing the conductivity. Moreover, the gas sensor performances of EG-intercalated samples are much higher than the pristine ones. It was indicated that 1 T-MoS_2_ is vital in improving the gas sensor performance of MoS_2_. Zong et al*.* [207] carefully controlled the amount of 1 T and 2H concentration by the annealing process of hydrothermally prepared MoS_2_. The higher the annealing temperatures, the smaller the amount of 1 T concentrations, as shown in Fig. 13d. The highest NO_2_ detection performance (sensitivity up to 25% under 2 ppm NO_2_, rapid detection time of 10 s and LoD of 25 ppb) was achieved by annealing MoS_2_ at 100 °C, where the ratio of 1 T/2H is 2:3. The NO_2_ gas sensor mechanism and performances of 1 T/2H-MoS_2_ are shown in Fig. 13e, f. The preceding results show that the gas detection capability of 1 T/2H-MoS_2_ could be boosted by controlling the heterophase, which brings new insights into transition-metal dichalcogenide gas sensors. A further investigation should be performed, especially with the utilization of in situ/*operando* spectroscopy, to essentially improve our current understanding of how each phase’s stability and contribution to the overall gas sensing properties of MoS_2_. Ideally, the papers report MoS_2_ gas sensors should be accompanied by DFT simulation to reveal the principle gas sensing mechanism.

### Surface Functionalization with Noble Metals

Numerous works on the noble metals-functionalized gas sensing materials have significantly enhanced responsivity, improved/tuning selectivity, and lowered working temperatures. As mentioned in the earlier discussion, pristine MoS_2_ has shown a promising gas sensing performance. However, it is accompanied by several limitations, including poor selectivity due to high cross-sensitivity to many gases and limited sensitivity at room temperature. Surface functionalization by noble metals has been applied to metal oxides gas sensing and non-oxides, including the MoS_2_. Noble metals, especially in nanoparticles (NPs) form, are utilized because they generally promote a more catalytic process via spill-over effect and electronic sensitization through charge carrier concentration and significantly alter internal electrical conductance or resistance of MoS_2_ measured by the sensing system. The catalytic reactions always follow the preceding gas adsorption/desorption process, despite the nature of gas (reducing or oxidizing) and gas composition (organic or non-organic). The spill-over effect by noble metals loading on the sensing material's surface helps lowering the potential energy dissociation of molecular oxygen (O_2_) in the air, so that ionization process into monoatomic O is facilitated. It also facilitates the ionized O transport to the MoS_2_ surface. The process cultivates the increase in the adsorbed oxygen ions on the materials for further reaction with tested analytes. The work function of noble metals is critical in regulating the mechanism, and herewith the modified gas sensing mechanism of MoS_2_ under different noble metals loading is discussed. The work functions of MoS_2_, Au, Pt, Pd, and Ag are 4.6, 5.1, 5.6, 5.4, and 4.8 eV, respectively. Due to the different work functions, in which the MoS_2_ has a lower work function than many noble metals, upon the contact, the electron will flow from MoS_2_ to noble metal through the depletion channel until the Fermi energy levels are equalized. Because of this process, the charge carrier concentration and mobility in depleted regions are improved and dissociated oxygen is more captured. More active interaction between ionized oxygens and the analytes is expected to improve gas sensing properties upon the analyte flow. The MoS_2_ surface decoration by noble metals can be performed using several approaches, including heat treatment, DC sputtering, chemical reduction, or directly adding the chemical reagent containing noble metals as the precursors under a one-pot synthesis condition.

**Fig. 14:**
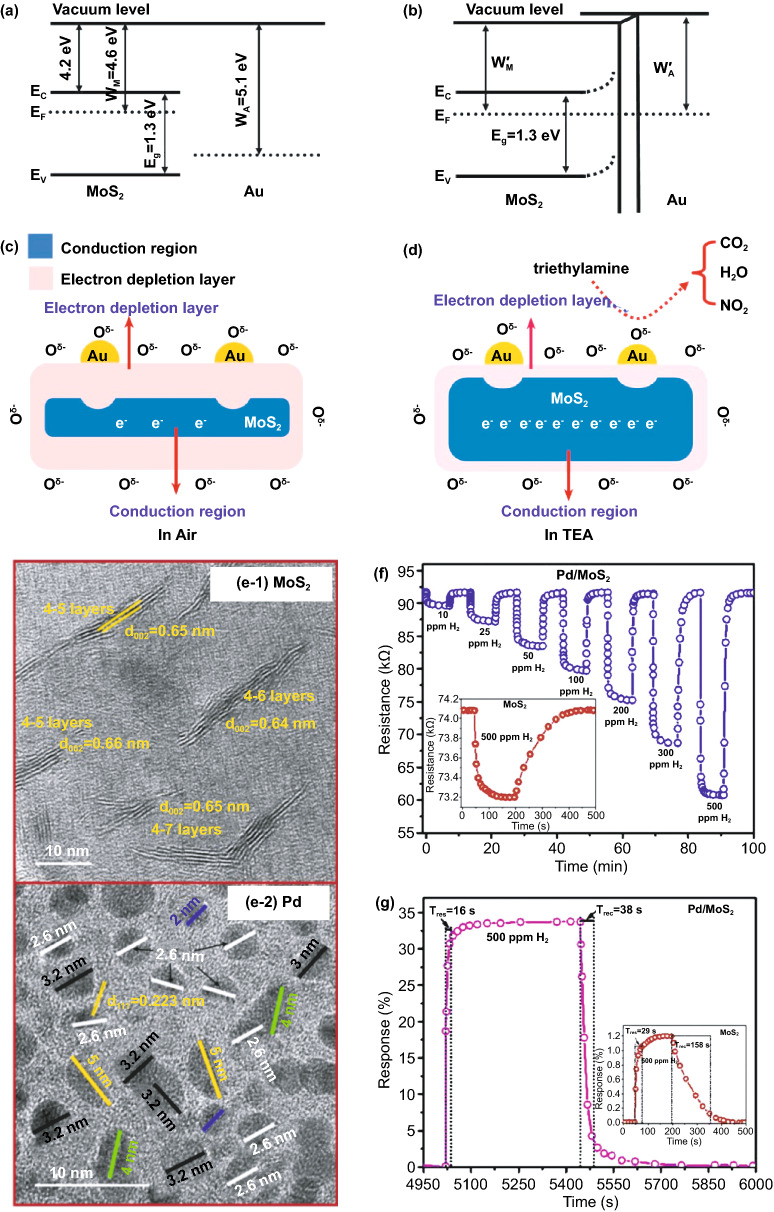
**a** Concept map of the energy band configurations for MoS_2_ and Au. **b** Energy band changing of Au@MoS_2_ heterojunction. **c, d** Schematic of material before and after gas sensing response. Reprinted with permission from Ref. [208]. Copyright, 2020, Elsevier B.V. HRTEM images of (**e-1**) MoS_2_ and (**e-**2) Pd nanoparticles. **f** A characteristic response curve (electrical resistance as a function of time) and **g** the sensor response curve of the Pd/MoS_2_ hybrid and the pristine MoS_2_ (inset) thin-film sensing devices toward H_2_. The gas sensing measurement was conducted at room temperatures. Reprinted from Ref. [212] with permission. Copyright, 2020, Elsevier B.V

It is noticed that the different noble metals will determine the different gas selectivity of MoS_2_ to some extent. For example, Au@MoS_2_ nanostructures can directly be grown on ceramic tubes in one-pot hydrothermal treatment at 180 °C. Au nanoparticle decoration was deposited by DC sputtering with a predesignated sputter times [208]. The Au nanoparticles have a spherical shape with a diameter of 5 nm. Au@MoS_2_ exhibited a remarkably higher response (5 times) and faster recovery speed to trimethylamine (TEA) gas at 280 °C than pristine MoS_2_. As shown in Fig. 14a–d, the band depletion occurred during the contact between MoS_2_ and Au due to the charge transfer process. The O_2_ was adsorbed and then dissociated to O^−^ on the Au surface before redistributing it onto the MoS_2_ surface. The thickening of the depletion layer and the increase in spilled O^−^ have increased the electrical resistance. During the TEA flow, the electrical decreased due to the active reaction of O^–^ with TEA and the removal of electrons in the depletion barrier. Au is believed to prefer adsorbing amine functional groups, as the Au–MoS_2_ had a remarkable sensing response towards ammonia [209].

Pd-functionalized MoS_2_ sensor acted differently from that of Au@MoS_2_ because it had shown excellent hydrogen gas sensing properties, *e.g.,* in the case of Pd-MoS_2_ nanosheets [210], Pd-MoS_2_ nanostructures [211], and vertically aligned edge-oriented MoS_2_ nanostructured thin film functionalized by Pd nanoparticles [212]. Figure 14e–g shows that the exfoliated MoS_2_ has 4–7 layered structures while Pd nanoparticles are 2–5 nm in size. Furthermore, Pd-functionalized vertically aligned MoS_2_ thin film has an average thickness of 19.5 nm. At RT, the sensor exhibited the highest response of 33.7% to 500 ppm of H_2_ with a rapid sensing response and recovery times (16/38 s). The spontaneous dissociation of hydrogen molecules on the Pd metals is the firm reason behind the strong response of Pd@MoS_2_, in which the formation of PdH_x_ affected the considerable resistance alteration. The distinguished selectivity behavior can also be found in the Ni-, Pt-, and Ag- loaded MoS_2_ nanostructures gas sensing [213–216]. However, the underlying mechanism of how the gas dissociation process occurs on the surface of noble metals is still uncertain. The comprehensive computational studies, such as combining DFT calculation and molecular dynamic (MD) simulation, are essential in the future to provide a deeper insight into the gas sensing mechanism in noble metals-functionalized MoS_2_.

### Elemental Doping

**Fig. 15:**
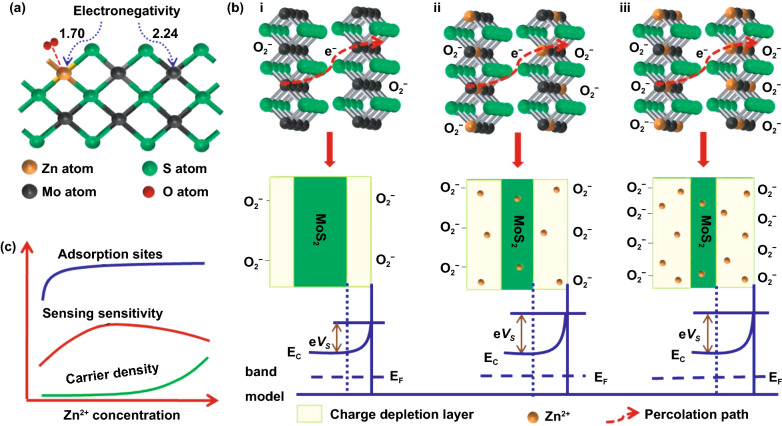
**a** Surface adsorption illustration of Zn: MoS_2_ UNs, **b** structural and band models showing the role of doping Zn^2+^, i: pure MoS_2_, ii: MoS_2_ with intermediate Zn^2+^ concentration, iii: MoS_2_ with high Zn^2+^ concentration, and **c** possible effects of Zn^2+^ concentration on absorption capacity, carrier density and sensing sensitivity. Reprinted from Ref. [221] with permission. Copyright 2018, Elsevier

Various atoms are suitable doping elements for improving the gas sensor performance of MoS_2_. Sulfur or molybdenum substitution by foreign atoms is expected to improve the gas adsorption ability of MoS_2_. As in many initial investigations, the introduction of doping into crystal structures of pristine MoS_2_ [217] aims to increase charge carrier transfer via band structures alignment, form more effective gas adsorption sites, or create a trapping mechanism for suppressing the electron–hole recombination. Pristine MoS_2_ is a natively n-type semiconductor originated from electron-donating sulfur vacancies, and the intrinsic n-type conductivity can be tuned to p-type conductivity with suitable substitutional atomic doping [218–220]. The resistivity behavior during the sensing mechanism will likely be similar to those of most metal oxides. Thus, discussion on the sensing mechanism has focused on the effect of dopings on gas sensing properties of MoS_2_ proposed by recent investigations. For instance, Zn doping is the effective dopant to induce p-type conductivity and tailoring effect on the MoS_2_ ultrathin nanosheets gas sensing properties. In this case, the Zn atom replaced the Mo atom at the edge site, inducing the formation of Mo vacancies and acted as a new adsorption site for both O_2_ and NO_2_ gas due to the difference in electronegativity (Zn^2+^: 1.70 and Mo^4+^: 2.24). Therefore, it is easier for oxygen molecules to capture the electron from the Zn site, resulting in enhanced adsorption capacity and wider depleted regions. However, when the Zn^2+^ amount reached above 5% state, the adsorption capacity gets saturated, potentially decreasing the gas sensing performance (Fig. 15**).** From this understanding, the choice of atomic doping and its amount are critical to tailor the gas sensing properties.

**Fig. 16:**
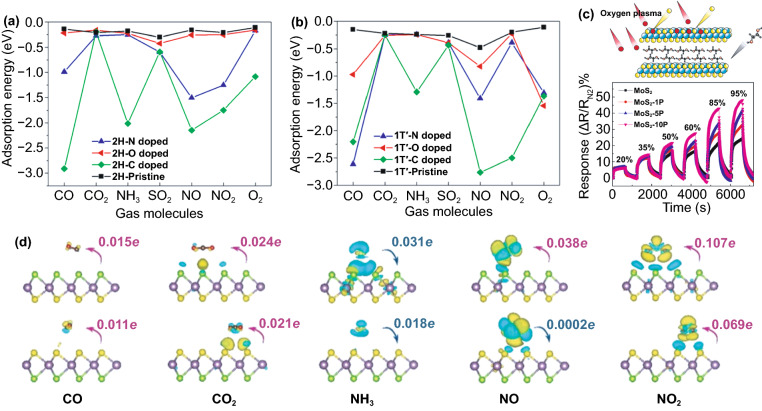
Adsorption energy of gas molecules on the C-, N-, O-doped **a** 2H- and **b** 1 T-MoS_2_ monolayers. Reprinted with permission from Ref. [133] Copyright 2020, American Chemical Society. **c** Sulfur substitution using O_2_ plasma irradiation of MoS_2_ and humidity sensing response values of MoS_2_ irradiated by O_2_ plasma. Reproduced with permission from Ref. [222]. Copyright 2020, American Chemical Society. **d** Charge density difference (CDD) for gas molecules adsorbed on the Se side (top panel) and S side (bottom panel). The yellow (cyan) region represents charge accumulation (depletion) and the isosurface. The orientation and the value of charge transfer of these molecules adsorbed on the Se and S surfaces are denoted. Adapted with permission from Ref. [229]. Copyright 2019 The Royal Society of Chemistry

Although there are limited reports of doped MoS_2_, recent theoretical calculations studies show that the gas adsorption ability of MoS_2_ can be enhanced by ion substitution with other atoms. For example, Linghu et al*.* [133] reported the theoretical investigation on the effect of S substitution of MoS_2_ by various nonmetallic atoms (C, N, and O) on the CO, CO_2_, NH_3_, SO_2_, NO, NO_2_, and O_2_ gases adsorption ability. The results show that the anions significantly improve the gases adsorption ability of both 2H-MoS_2_ and 1 T’-MoS_2,_ as shown in Fig. 16a, b. The adsorption energy of anion-doped MoS_2_ samples shows a massive improvement than pristine MoS_2_. Compared to other doping elements, C-doping shows the best adsorption ability for all tested gases. For 1 T’-MoS_2_, the N-doped MoS_2_ and O-doped MoS_2_ showed the best adsorption ability on CO_2_ and O_2_, respectively. Other reports also have confirmed that N-doping, O-doping, and C-doping MoS_2_ improve the gas adsorption performance of MoS_2_, directly affecting the gas sensor performance. Recent experimental evidence of the improvement of MoS_2_ gas sensor by the presence of O atom was reported by Taufik et al*.* [222] O_2_ plasma treatment was used to introduce O atom into the crystal structure of MoS_2_ for enhancing the humidity sensor performance of MoS_2,_ as shown in Fig. 16c. The more extended O_2_ plasma irradiation led to more oxygen amount in the crystal structure. O atom's presence significantly improved the humidity sensor performance of MoS_2_ in the crystal structure. Although the direct evidence of the presence of N-doped MoS_2_ and C-doped MoS_2_ for gas sensor devices had not been reported, the N-doped MoS_2_ and C-doped MoS_2_ are widely used for hydrogen evolution reaction and photocatalyst [223–226]. According to Li et al*.* [225], the insertion of N atoms induced the defect on S sites. N atoms optimize the electron density beneficial for hydrogen evolution reactions. Guo et al*.* [227] reported that the edge of the MoS_2_ structure can be engineered by the presence of N-doping and increase the hydrogen evolution reaction with a low overpotential of 114 mV to produce a current density of 10 mA cm^−2^ and high stability. The edge is also essential in the gas sensor performance because the active sites of MoS_2_ are primarily located at the edge of its layer.

Apart from the anions mentioned above, other chalcogen anions, such as selenium and tellurium, are potential doping sources for modifying the structure of MoS_2_. The advantage of using chalcogen anions as dopant is the structural similarity with MoS_2_ [228]. Therefore, the MoS_2_ structure is easily modified without additional impurities and secondary phase. According to Jin et al*.* [229], the MoSSe Janus structure might improve the gas adsorption properties of MoS_2_. Figure 16d shows the adsorption parameters of MoS_2_, MoSSe, and MoSe_2_ on various gas adsorption, including CO, CO_2_, NO, NO_2_, and NH_3_ [229]. The adsorption properties of S- and Se-modified MoSSe surfaces were investigated. The results showed that in the CO and CO_2_ gas, the adsorption distance between MoSSe and analyte was greater than 3 Å. This shows that the adsorption process is weak (physisorption). In NH_3_, NO, and NO_2_ gas, the adsorption distance is less than 3 Å, which is considered strong adsorption (chemisorption). The adsorption distance between MoSSe and adsorbed molecules/gases is closer than MoS_2_ and MoSe_2_. The E_a_ values (magnitudes) of all the studied molecules adsorbed on the Se-layer were obviously larger than those on the S-layer, indicating the surface selectivity of Janus MoSSe for these molecules. Therefore, gas molecules need to be adsorbed on the Se surface with higher binding strengths. Furthermore, the E_a_ values of NH_3_ and NO_2_ adsorption on the Janus layer were relatively larger, leading to higher selectivity of the MoSSe structure. There are several interesting phenomena to be considered in this regard. For instance, CO, CO_2_, and NO_2_ on the MoSSe act as the charge acceptors while the NH_3_ molecule behaves as the charge donor to the Se or S side of the monolayer. Particularly, NO acts as an acceptor on the Se side and as a donor on the S side. Figure 16d shows the charge density difference between MoSSe samples and adsorbed gases. The obvious charge redistribution occurred in NH_3_, NO, and NO_2_ gases which were considered as sensitive molecules to be adsorbed by MoSSe structures. The results have demonstrated that the modification of MoSSe has relatively more potential to improve molecular adsorption ability.

Besides the anion doping, modification of MoS_2_ structures-led enhanced sensing performances can be conducted by cations elements. Zhu et al*.* [230] established that Nb, V, and Ta doping into MoS_2_ monolayer significantly improves the gas adsorption properties to CO, NO_2_, H_2_O, and NH_3_ molecules. This effect occurs due to the substantial overlap between the metal and orbitals and gas molecule orbitals, leading to activation of the adsorbed gas molecules. Analysis of Bader charge shows that more charge transfer (−0.66 e^−^ to −0.72 e^−^) occurs from metal (V, Nb, Ta)-doped monolayer MoS_2_ to the oxidizing gas molecules (NO_2_) acting as acceptors. Regarding CO molecules adsorption, relatively fewer electrons (about − 0.24 e^−^−0.35 e^−^) transfer occured from the substrate to the adsorbed gases. In experimental works, the MoS_2_ gas sensing performances have been successfully modified via Zn, Co, Ni, and Fe atomic dopings. These atoms usually replace the Mo atom due to similar cationic behavior. Shao et al*.* [221] varied the Zn concentration in MoS_2_ structures to understand the optimum gas sensing performances optimum condition. Regardless of the tested analytes, the 5%-Zn-doped MoS_2_ attained the highest gas sensor. Zhang et al*.* [231] used Co, Ni, and Fe as dopants sources for MoS_2_. The improved SO_2_ gas sensor performance was observed in the Co-, Ni-, and Fe-doped MoS_2._ Compared to other cations, Ni-doped MoS_2_ exhibited the best SO_2_ gas sensor performance. DFT calculation showed that the cations-doped MoS_2_ increases the adsorption energy, decreases the adsorption distance, and increases the charge transfer process between MoS_2_ and SO_2_. Moreover, Ni-doped MoS_2_ showed the highest adsorption energy, closer adsorption distance, and highest charge transferability. All these results support the anion and cation doping process of MoS_2_ that modifies the crystal structure and increases the gas adsorption performance of MoS_2_. However, the long-term and phase stability of the anion incorporation into MoS_2_ crystal structures against environmental oxidation are lack of detailed studies.

### Heterostructures Coupling

When two dissimilar materials are in contact, heterointerfaces, commonly known as heterojunction, are formed. It offers various advantages to the improvement of many gas sensing materials. The underlying enhanced mechanisms of heterostructures include (i) band structures alteration due to Fermi level adjustment, (ii) depletion layer enlargement, (iii) synergistic surface reaction via electronic sensitization, and (iv) catalytic promotions [28]. Therefore, heterostructures coupling arose as one of the advanced strategies for optimizing gas sensing performances of MoS_2_. Many materials have been recently combined with MoS_2_ to achieve good gas sensors materials, such as carbon-based materials, oxide materials, and other TMDs materials.

**Fig. 17:**
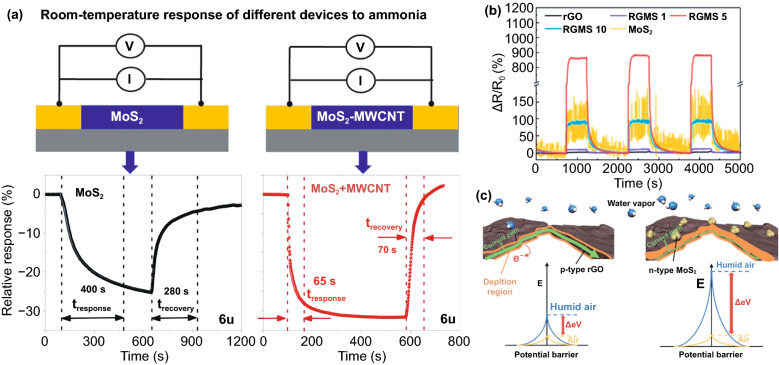
**a** NH_3_ sensor performance of MoS_2_ and MoS_2_/carbon nanotubes composites Reproduced from Ref. [232]. Copyright 2020, Elsevier. **b** Response curves of rGO, RGMS 1, RGMS 5, RGMS 10, and MoS_2_ to 50% RH at room temperature. **c** Schematic of the mechanism with enhanced humidity sensing properties of the enhanced depletion region on bare rGO and RGMS. Reproduced from Ref. [233]. Copyright 2018, The Royal Society of Chemistry

The combination between MoS_2_ and carbon-based materials has been widely investigated due to the synergistic effects between the good sensitivity of MoS_2_ with the good conductivity and high specific surface area of carbon-based materials. For instance, Sing et al*.* [232] reported the formation of MoS_2_/CNTs heterostructures to detect NH_3_ gas at RT. The addition of CNTs into MoS_2_ increased the specific surface area. The fabricated sensor devices based on MoS_2_ and MoS_2_/CNTs illustrated in Fig. 17a exhibited the n-type semiconducting behavior and showed room-temperature NH_3_ detection down to 12 ppm-level. Regarding MoS_2_, the corresponding response time (t_*res*_ = 400 s) and recovery time (t_*recov*_ = 280 s) are very large with LoD down to 1.2 ppm. In comparison, the prepared MoS_2_/CNTs exhibited faster response–recovery (65 and 70 s, respectively) features along with enhanced relative response for various ammonia concentrations, ranging from 12 to 325 ppm. The improvement of ammonia detection performance of MoS_2_/CNTs is attributed to the higher adsorption energy of MoS_2_/CNTs than MoS_2_ for ammonia adsorption.

The 2D/2D heterojunction showed fascinating effects on the gas detection improvement governed by large and strong interface contact areas. This is due to the close face-to-face contacts between 2D layered materials. The combination of 2D MoS_2_ with 2D graphene-based materials needs to facilitate stronger interfacial electrical coupling and charge transfer than 0D/2D, 1D/2D, and 3D/2D. Park et al*.* [233] reported the successful formation of MoS_2_/RGO composites for water vapors sensing devices with fast response, excellent selectivity, and ultrahigh sensitivity based on 2D rGO and 2D MoS_2_ hybrid composites (RGMSs). The RGMSs were fabricated by simple ultrasonication without the addition of additives and additional heating. Compared to pristine rGO, the RGMS exhibited a 200 times higher response to water vapors at RT. The significant enhancement in the sensing performance of the composite was attributed to electronic sensitization due to p–n heterojunction formation and porous structures between rGO and MoS_2_, as shown in Fig. 17b, c. The synergistic combination of rGO and MoS_2_ could be applied to construct a flexible humidity sensor. Besides, a recent study suggested that carbon dots (CDs) can modify the humidity sensing properties of MoS_2_ nanosheets because of the abundant surface functional groups of CDs that can possibly adsorb water molecules stronger than the bare MoS_2_ [234]. Yue et al*.* [235] investigated the formation of graphene/MoS_2_ quantum dots composites for NH_3_ and NO_2_ gas recognition. The NO_2_ detection gives a negative response value, while the NH_3_ detections have a positive response value attributed to the different charge transfer mechanisms between NO_2_ and NH_3_. In NO_2_ gas detection, all sensor materials lose the electron and increase the resistance. In NH_3_ detection, the sensor materials gain an electron from NH_3_ due to the electron donor properties of NH_3_. These results confirm that the combination of MoS_2_ and carbon-based materials improve gas sensor performance.

**Fig. 18:**
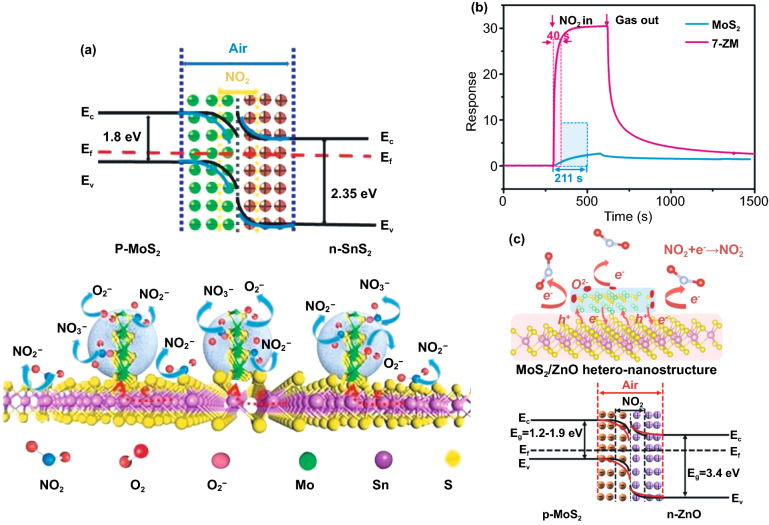
**a** Schematic of sensing mechanisms of MoS_2_/SnS_2_ samples the sensors to NO_2_. nanocomposite. Reprinted with permission from Ref. [236]. Copyright 2020 Elsevier. **b** Response and recovery curves of MoS_2_ NSs and 7-ZM at 5 ppm NO_2_. **c** Schematic of sensing mechanisms of MoS_2_/ZnO hetero-nanostructures to NO_2_ molecules and MoS_2_/ZnO hetero-nanostructures energy band structure in Air and a NO_2_ atmosphere. Reprinted with permission from Ref. [238]. Copyright 2018 American Chemical Society

Having many resemblances in term of crystal structures, combining MoS_2_ with other TMDs families provide more synergistic process and component suitability, which often increases the gas sensor ability. An assemble heterostructure containing MoS_2_ and SnS_2_ composite has been successfully fabricated by Liu et al*.* [236] using a hydrothermal approach. The MoS_2_/SnS_2_ composite exhibited an outstanding improvement for NO_2_ gas detection compared to MoS_2_ and SnS_2_. The higher NO_2_ sensing ability of MoS_2_/SnS_2_ composites is attributed to the p–n junction formation. In the p–n heterojunction system, the electrons flow from n-type SnS_2_ to p-type MoS_2_. Consequently, electron depletion layers formed on the surface of SnS_2_. Simultaneously, the holes from MoS_2_ tend to diffuse to the surface of SnS_2_, which leaves a negatively charged region. Electron–hole diffusion continues until the Fermi level of the composite reaches an equilibrium state. The barrier at the SnS_2_/MoS_2_ interface and the cumulative layer on the surface of the MoS_2_ contribute to the low conductivity of the SnS_2_/MoS_2_ nano-heterostructures in air, confirmed by I–V results. However, in the fresh air, O_2_ is adsorbed on the surface of the sensor and changes into O_2_^−^. When the sensor is exposed to NO_2_ gas, the molecules are adsorbed on the surface of the sensor and capture free electrons from the accepter level of the sensor to form NO_2_^−^. Also, the NO_2_ molecules reacted with chemisorbed oxygen and consequently converted into NO_3_, disturbing the electric field's equilibrium to decrease the barrier width and increase the sensor conductivity toward NO_2_ gas [236]. The entire process is simplified in Fig. 18a. Ikram et al*.* [237] demonstrated the synthesis of a heterojunction of few-layer MoS_2_ nanosheets (NSs) with multilayer WS_2_ using a simple one-pot hydrothermal process. They successfully improved the gas sensing performance of TMD heterostructure nanomaterials (NMs) for NO_2_ at room temperature. The response value of MoS_2_ and MoS_2_@WS_2_ with Mo: W atomic ratio of 3.8:1 (MWS-1), 1.55:1 (MWS-2), and 0.36:1 (MWS-3). The NO_2_ detection response of all composite samples was higher than MoS_2_ samples. Similarly, the response and recovery processes during the NO_2_ adsorption are faster than MoS_2._ The commendable selectivity and appreciable stability to NO_2_ gas are believed to be a synergistic effect between MoS_2_ and WS_2_ NSs originating from the enhanced surface area and remarkably increased exposed active sites for NO_2_ adsorption.

MoS_2_ has interesting features to support the performances of oxide-based sensing materials. One major problem of oxide-based is related to the high operating temperature of the oxide materials due to low conductivity. To make oxide-based materials applicable in the room-temperature regime, MoS_2_ is needed because of its relatively good conductivity and being reactive at room temperature for diverse types of gases. Han et al*.* [238] demonstrated MoS_2_/ZnO heterostructure's formation for improving NO_2_ sensing performance. The purpose of this heterostructure formation is to fabricate the p–n heterostructures as an effective way to modulate the intrinsic electronic properties of MoS_2_ nanosheets (NSs), achieving high sensitivity and excellent recovery properties. Figure 18b shows the comparative NO_2_ response between pure MoS_2_ and MoS_2_/ZnO (7-ZM). The 7-ZM displays superior performance with an excellent response of 30 (R_a_/R_g_) to 5 ppm NO_2_ with a fast response time of 40 s and outstanding recovery ability. Figure 18c shows a graphical illustration of the NO_2_ sensing process. During the NO_2_ adsorption, the electron from MoS_2_ and ZnO tends to move toward NO_2_ molecules. Holes accumulate at the surface of MoS_2_ NSs, and the width of the heterojunction barriers is decreased. Therefore, the conductivity of MoS_2_/ZnO heterostructures greatly increases, contributing to the enhanced response values. Constructing p–n hetero-nanostructures for 2D materials is a versatile solution for achieving excellent sensing performances. According to Wang et al*.* [239], the combination of MoS_2_ and SnO_2_ effectively improves gas sensor performance. In this study, the NH_3_ sensing performance of MoS_2_/SnO_2_ at RT was examined. The NH_3_ sensing performance of MoS_2_/SnO_2_ is much higher than MoS_2_ and pure MoS_2_. They also suggested the improvement of the NH_3_ sensing performance of MoS_2_/SnO_2_ is due to the formation of n–n junction between MoS_2_ and SnO_2._ In a summary, heterojunction fabrication between MoS_2_ and other materials, including carbon-based, TMDs, and oxide materials, has exceptional gas sensing benefits due to the advantages of the interfacial charge transfer mechanism. The computational dynamic simulation may give a deeper understanding of the hole–electron mobility and transfer at the interface, especially during the gas adsorption.

### Other Recent Strategies

#### Enhancement by Light Irradiation

**Fig. 19:**
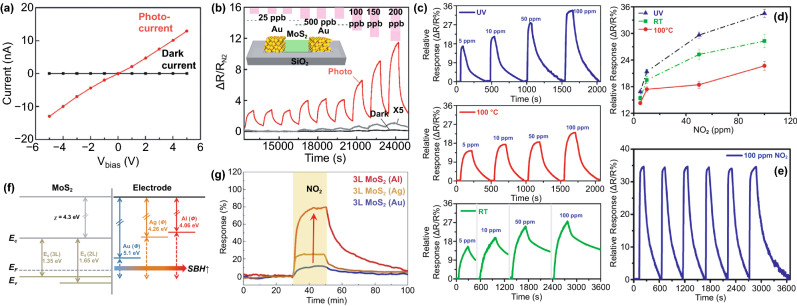
**a** I–V dependence of the Au–MoS_2_–Au device in the dark and under red LED illumination with incident power of 60.9 nW. **b** Effect of NO_2_ gas exposure at concentrations from 25 to 200 ppb on normalized resistance of the Au–MoS_2_–Au device in the dark (black line; gray line shows fivefold magnified data) and under red LED illumination (red curve). Reproduced from Ref. [240] with permission. Copyright 2019, American Chemical Society. **c** Transient relative response of sensor to 5, 10, 50, and 100 ppm concentration of NO_2_ at room temperature (RT), at 100 °C, and at RT under UV illumination (1.2 mW cm^−2^). **d** Relative response versus NO_2_ concentration at RT, 100 °C, and at RT under light. **e** Cyclic test to 100 ppm of NO_2_ at RT under UV light. Reproduced from Ref. [241] with permission. Copyright 2017, American Chemical Society. **f** Band diagram of MoS_2_ with metal electrodes. **g** Sensing characteristics of NO_2_ for 3L MoS_2_ with Al, Ag, and Au electrodes. Reproduced from Ref. [243] with permission. Copyright 2019, American Chemical Society

The recent experimental results have shown that light irradiation effectively increased the gas sensor performance of MoS_2_. The light irradiation can have several impacts on the MoS_2_ surface, which will benefit gas sensor enhancement. The electron–hole formation is unavoidably existed during the light irradiation due to its small band gap of MoS_2_. The increase in the charge carrier formation during light irradiation could improve the sensor response due to increased reaction probability between the charge carrier and gases. Moreover, light irradiation can remove the oxygen ion from the surface, which will be beneficial to increase the reactivity of the tested gas with the MoS_2_ surface. Pham et al*.* [240] have investigated the NO_2_ sensor performance of MoS_2_ by using red-light irradiation. As mentioned earlier, light irradiation can increase the charge carrier concentration. The increase in the charge carrier concentration directly relates to the increase in the conductivity, as shown in Fig. 19a. The I–V characteristics increase about 500% after light irradiation, which indicates this material is light sensitive. The MoS_2_ was deposited on the SiO_2_ substrate through spin-coating techniques, and gold was used as an electrical channel. Figure 19b shows the NO_2_ response under dark and light irradiation (inset is MoS_2_-based sensor device). The NO_2_ detection performance of MoS_2_ significantly improved after light irradiation and showed extremely high sensitivity to ppb level NO_2_ gas exposure up to 3.3% ppb (3300% ppm) and sub-ppb limit of NO_2_ gas detection at the 0.1 ppb level. Another report from Kumar et al*.* [241] has also shown the improvement of the NO_2_ sensor of MoS_2_ through UV-light irradiation. Figure 19c–e shows the gas sensor performance of MoS_2_ under light irradiation, heating treatment, and room temperature. The sensor response of UV-activated is higher than at room temperature and with annealing treatment. Moreover, the response and recovery speed time are greatly improved under UV-light irradiation. The increase in the response and recovery speed under UV-light irradiation is due to the substantial enhancement in response to full reproducibility of multilayer MoS_2_ gas sensor to NO_2_ gas at room temperature under the UV illumination was attributed to the removal of contamination from the surface (clean surface, renders greatest possible reactive sites per unit volume) and the minor effect of photogenerated electrons in the conduction band of MoS_2_.

#### Substrate Engineering

The electrode preparation is also crucial in designing suitable sensor devices because the different substrates and different electrical channels will give a different electrical signal response. Ali et al*.* [242] have investigated the detailed preparation of the electrode for gas sensor measurement of MoS_2_. The different substrates and also different channel lengths have been carefully conducted. Two kinds of substrate (SiO_2_ and h-BN) have been used as a substrate. In MoS_2_/h-BN, a sharp decrease in mobility is observed for low concentration gas exposures, which can be explained by the increase in scattering sites on adsorption of NO_x_ molecules due to the device being more homogeneous on a flatter substrate. In the case of the MoS_2_/SiO_2_ device, the change in mobility in NO_x_ presence is much lower for low concentrations, which shows that influence of surface roughness is more dominant. The different channel length is also crucial for the device with a shorter channel length shows a relatively higher response than a long channel, as the charges undergo less scattering during transport through a shorter channel. Another report from Kim et al. [243] has investigated the difference in channel materials. Three different metals were used. Al, Ag, and Au were used as conductive material for the electrical channel. The use of different conductive materials can change the Schottky barrier height (SBH) due to the different metals' work functions, as shown in Fig. 12f. This Schottky barrier height also affects the gas sensor response. Figure 12g shows that the NO_2_ detection performance improves with the lower work function. The electrode with a low work function increased the responsivity.

Each advanced approach has interesting benefits for sensing enhancement. The precise selection and sensor design greatly produce sensors with expected performance and more effective experimental time. To clarify each strategy’s contribution, all reviewed strategies to advance the gas sensing performance of MoS_2_ are summarized in Table 2.

**Table 2:** Improvement strategies of MoS_2_-based gas sensor

Improvement strategies	Sensing materials	Target gas	Conc. (ppm)	T (°C)	Sensitivity	Response/recovery times (s)	Refs.
Morphological design	Hierarchically MoS_2_ nanospheres	CO	500	230	92.6(*R*_*a*_*/R*_*g*_)	18/15	[188]
	3D hierarchical porous MoS_2_ microspheres	H_2_	500	120	20.5%	30/60	[185]
	MoS_2_ nanoflakes	Ethanol Methanol	10	50	17.6(I_D_/I_0_) 10.8(I_D_/I_0_)	53/– 67/–	[244]
	3D MoS_2_ Aerogel	NO_2_	0.5	200	120%	33/107	[195]
	Edge-oriented MoS_2_ flakes	H_2_	10,000	RT	1%	14.3/136.8	[189]
	Atomic layered MoS_2_	NO_2_	0.12	RT	35%	*n.a*	[193]
	Monolayer MoS_2_	Triethylamine	10	RT	18%	5/5	[190]
	Single- and Multilayer MoS_2_	NO	2	RT	80%	*n.a*	[191]
	Monolayer MoS_2_	NO_2_ NH_3_	0.02 1	RT	20% 40%	n.a	[194]
Phase control Surface functionalization	10%1 T Mo_0.87_W_0.13_S_2_	Acetone	100	RT	1.6%	n.a	[206]
	30%1 T Mo_0.87_W_0.13_S_2_	Acetone	1000	RT	0.4%	n.a	[206]
	1 T/2H MoS_2_	NO_2_	2	RT	25%	10/700	[207]
	1 T/2H (1.7) MoS_2_	Toluene	100	RT	12.50%	52/48	[172]
	1 T/2H (4) MoS_2_	Toluene	100	RT	16.29%	52/26	[172]
	Au MoS_2_	NH_3_	1000	60	9.6 (*R*_*a*_*/R*_*g*_)	*n.a*	[209]
	Au MoS_2_	Triethylamine	50	280	59 (*R*_*a*_*/R*_*g*_)	*n.a*	[208]
	Pd-MoS_2_	H_2_	10,000	RT	35.3%	786/900	[210]
	Pd-MoS_2_	H_2_	50,000	RT	10 (*R*_*a*_*/R*_*g*_)	83/-	[211]
	Pd-MoS_2_	H_2_	500	RT	33.7%	16/38	[212]
	Ni-MoS_2_	H_2_S	2	RT	80%	*n.a*	[215]
	Pt-MoS_2_	NH_3_	70	RT	36%	*n.a*	[213]
	Pt-MoS_2_	H_2_	100	150	10 (*R*_*a*_*/R*_*g*_)	4/19	[214]
	Ni-MoS_2_	H_2_S	2	RT	80%	*n.a*	[215]
	Ag-MoS_2_	Methanol	100	RT	21.6%	*n.a*	[216]
Elemental doping	O-doped MoS_2_	H_2_O	RH (95%)	RT	47%	228/184	[222]
	Zn-doped MoS_2_	O_3_	0.6	RT	8%	5.5/10.1	[221]
	Fe-doped MoS_2_	SO_2_	500	RT	5%	60/107	[231]
	Ni-doped MoS_2_	SO_2_	500	RT	14%	54/92	[231]
	Co-doped MoS_2_	SO_2_	500	RT	4%	58/98	[231]
Heterostructures	MoS_2_/rGO	H_2_O	RH (85%)	RT	2494.25%	6.3/30.8	[233]
	MoS_2_ WS_2_	NO_2_	50	RT	25%	2/36	[237]
	MoS_2_/ZnO	NO_2_	5	RT	3050%	*n.a*	[238]
	MoS_2_/SnO_2_	NH_3_	50	RT	90 (*R*_*a*_/*R*_*g*_)	2.3/1.6	[239]
	MoS_2_/SnO_2_	NO_2_	5	RT	18.7 (G_g_/G_a_)	74/–	[245]
	MoS_2_/Carbon Dots (CDs)	H_2_O	RH (15–80%)	RT	0.5 (I_R_/I_0_)	22/71	[234]

*n.a.* = data not available

## Other Molybdenum-Based Gas Sensor Materials

Concerning α-MoO_3_ and MoS_2_, other molybdenum-containing compounds have been recently examined for their functionality as next-generation solid-state chemiresistive gas sensing materials with desired specifications. MoSe_2_ and MoTe_2_ are in the same family of TMDs, similar to MoS_2_, and having two-dimensional layered structures with a high aspect ratio [246]. The physical and electronic properties of MoSe_2,_ such as very narrow band gap (1.1 eV for bulk and 1.5 eV for monolayer), good full-spectrum absorption at 200–800 nm, low internal resistance, and high carrier mobility (100 cm^2^ V^−1^ s^−1^), support its utilization in optoelectronic and photocatalysis application [247, 248]. Specifically, 2D structures manifested by MoSe_2_ secures the ultra-large specific surface area and abundant surface adsorption sites that govern the gas sensing performances. Because of these properties, MoSe_2_ exhibited excellent sensing performances to sensitively recognize harmful and toxic gases, such as NH_3_, NO_2_, CO, and H_2_S with LoD at ppb level and fast response/recovery times within few seconds [249–252]. 2D MoSe_2_ nanosheet can be synthesized via a liquid exfoliation approach in which the process is assisted by anhydrous ethanol as dispersant. With only a few layered structures, the 2D MoSe_2_ exhibited the improved detection to NO_2_ gas greater than bulk MoSe_2_ [253]. Advanced strategies have also been conducted to improve the gas sensing performance of MoSe_2,_ including noble metals functionalization (Au, Pd), morphology and structural control, and nanocomposites [249, 251, 254, 255]. MoSe_2_ is expected to have a bright prospect in gas sensing in the future. Although there have been extensive studies on MoS_2_ and MoSe_2_, there is still a lack of relevant research on MoTe_2_ gas sensing properties despite their equivalent structures. MoTe_2_ has a possible use for environmental monitoring, as initially suggested by Lin and group [256], followed by a few experimental works. MoTe_2_ demonstrated gas sensing ability to detect as low as 3 ppb of NH_3_ gas upon UV-light illumination. Due to the excellent MoTe_2_ electronic properties, UV light improved NH_3_ detectability [257]. Wu et al*.* stated that the MoTe_2_ sensing response behaved like a p-type semiconductor. With a similar approach, UV-light-illuminated MoTe_2_ gas sensors detected NO_2_ and ketones with high selectivity [258, 259]. The light-tunable sensing approach is a facile strategy and key performance applied in sensing platforms based on other 2D materials. Due to many structural similarities, the enhanced gas detection performances of MoSe_2_ and MoTe_2_ can be expected using approaches performed to MoS_2_. Though less pronounced, molybdenum carbide (*α*-MoC_1−x_ and *β*-Mo_2_C) nanoparticles showed unprecedentedly high signal-to-noise ratio (SNR) with the ability to detect the ppb levels of NH_3_ and NO_2_ [260]. Furthermore, its chemical stability and high melting temperature properties are suitable for sensing hazardous gases in a harsh environment, which cannot be achieved by oxides semiconducting gas sensor. Hence, the research utilizing other kinds of molybdenum-based sensors is highly encouraged to extend the future high-performance gas sensing materials.

## Summary and Future Challenge

Extensive studies on molybdenum oxides and dichalcogenides show a significant technological prospect and tremendous assets for multiple functional applications on the environment, energy, and health. Due to excellent and many interesting properties, including 2D layered structures, studies have examined the ability and feasibility of *α*-MoO_3_ and MoS_2_ as gas sensing materials. Various advancement strategies of *α*-MoO_3_ and MoS_2_ gas sensors have comprehensively been summarized. Regarding pristine *α*-MoO_3_ and MoS_2_, enhancement strategy was performed by morphological design and shape control, including 0D (quantum dots), 1D (monolayer nanosheet or nanoplates), 2D (nanorods, nanotubes, nanofibers, nanobelts), and 3D hierarchical structures (microspheres, microflowers, hollow nanostructures) to enlarge their surface area in order to allow more gas adsorption/desorption process and catalytic reactions. Particularly, intrinsic crystal defects in α-MoO_3_, such as oxygen vacancy formed after synthesis, provides a highly active site for molecular oxygen adsorption. Similarly, most of the active surface of MoS_2_ is situated at the edges of their layered structures. Therefore, it is essential to have phase control synthesis (1 T, 2H, and 3R) in bare MoS_2_ to ensure adsorbed oxygen molecules are exposed to their edges-faceted surface. Further effective strategies involve extrinsic chemicals or compounds, either surface functionalization, elemental dopants, and heterostructure coupler. Surface functionalization lowers the activation energy of oxygen dissociation, leading to more abundant ionized oxygens. However, the decorative surface thickness needs to be controlled to avoid blocking oxygen diffusion into sensing materials. Dopants are used to modify electronic, efficient, and crystal structures, mainly for band gap tuning, charge carrier sensitization, and defect formation. The option to heterostructures coupled-α-MoO_3_ and MoS_2_ is more pronounced to effectively improve gas sensing properties due to widely available developed compounds (other semiconductor ceramics, metals, or polymer) meant for a particular purpose such as extreme environment resistant and flexible/wearable sensors.

The combination of the above strategies can be developed with special attention to their methodological simplicity and effectiveness. In the case of α-MoO_3_, this oxide is very suitable for detecting VOCs, especially VOCs containing amine, due to its acid properties. However, its strong interaction results in a very long recovery time. Until this moment, the available solution is to apply a heating pulse at a relatively high temperature. This strategy is not suitable for α-MoO_3_ which can only be operated at low temperatures. Moreover, it cannot be applied to the oxide that is prepared at low temperature due to its properties that tend to change at high temperature, leading to affect its stability. Therefore, it is important to maintain the long-term performance by lowering the operating temperature. Another worth trying strategy is combining the oxide with basic materials such as ZnO. Furthermore, combined with carbon nanotubes (CNT) is can also be done because its report is still rarely be found. Similarly, the gas sensing properties of pristine MoSe_2_ and MoTe_2_ can be dramatically tailored by such approaches. Table 3 shows the advantages and disadvantages of each respective strategy.

**Table 3:** Advantages and disadvantages of each improvement strategy

Strategy	Advantages	Disadvantages
Morphological design	Versatile to obtain nanostructured materials Inexpensive equipment	Surfactant impurity Gas sensing performance cannot be easily predicted
Noble metal functionalization	High catalytic properties of noble metals to organic and non-organic compounds offers faster redox reaction, lead to rapid and high responsivity Reduce working temperature	Resource scarcity High cost Metal toxicity
Phase control	Effective and efficient to highly adsorb analyte by increasing the edge site	Issue on phase stability at certain temperature
Elemental doping	Improve charge carrier concentration Oxygen deficiency induced by charge compensation can be active sites for gas adsorption	Secondary impurity phases Morphology may be changed after doping due to crystal lattice adjustment
Heterostructures	Enhance electron–hole spatial separation More adsorption sites in heterojunction	Requires multistep synthetic approach which means more time and resource consuming In some cases, optimized working temperature increases

Despite enormous strategies for optimizing gas sensing properties of α-MoO_3_ and α-MoS_2_ developed until today, there is still a need to focus on and address some obstacles and challenges. Gas sensors are technologically important in modern society and help control atmospheric pollutions and their exposure to the environment or monitor human health. With the massive growth of information technology and Internet-of-Thing (IoT), the gas sensing research on 2D layered structured materials, including α-MoO_3_ and MoS_2_, can be integrated into a flexible and wearable sensor to provide real-time gas detection and point-care. For wide deployment, gas sensor device requires ultralow power utilization, low-cost fabrication, high signal-to-noise ratio, long time span, flexibility, and wearability on integrated electronic circuit and miniaturization. Achieving ultralow power consumption is still a critical task because the semiconducting properties of α-MoO_3_ require an external heat source to optimize their gas sensing performance, a similar case in MoS_2_. Although some works reported that α-MoO_3_ and MoS_2_ could work at room temperature, the sensitivity is still too low with terribly slow responses. The surface functionalization by noble metals may significantly reduce working temperature and, at the same time, improves the gas sensitivity and creates high-cost sensing devices due to resource shortage and high price. Because of high abundancy, carbon-based may potentially replace noble metals. Employing graphene oxides to ZnO microwires enabled the sensor device to work at room temperature with ultralow consumption [261]. Fabrication of *α*-MoO_3_- and MoS_2_-based sensors with ultrahigh signal-to-noise ratio is highly challenging. The ambient environment's intervention, such as humidity or interference gas, leads to high noise background and cross-sensitivity, reducing a “real” electrical signal generated by the tested gases. Therefore, this issue needs to be tackled to produce a highly selective gas sensor.

To realize the room-temperature sensor device, the humidity factor is very crucial to be considered as the major factor because our air consists of different humidity in different situations. If the sensor is stable under different humidity, the sensor is promising as a room-temperature device; however, if the sensor response is greatly altered during the humidity change, the response value is not reliable. Recently, researchers are still struggling with stabilizing the sensor response of MoS_2_ in a humid environment. Since the MoS_2_ is hydrophobic, it is very sensitive to react with water, altering the sensor response. High humidity decreases sensor response decrease. The decrease in the sensor response during the humid environment is due to the competition between water molecules and targeted gas interact with MoS_2_ surfaces. In compensation, MoS_2_ can be used as a humidity sensing material in practical application.

There is a huge demand economically and environmentally for sensor devices that can retain their sensing properties over thousands of repeated cycles to avoid added recycling costs and electronic waste (e-waste). The stability of *α*-MoO_3_- and MoS_2_-based sensors has been achieved for several days, but the measurement was conducted in a laboratory environment. The real test in the various environmental conditions, such as in winter and summer periods, needs to be performed to observe the environmental effect on gas sensing performance. Integration sensing materials and miniaturization into a flexible electronic circuit are fascinating areas for study, yet still far from development. Given layered structures with excellent mechanical properties, *α*-MoO_3_ and *α*-MoS_2_ need to provide high and homogeneous coverage on the interdigitated electrode of a flexible electronic substrate. The feasibility of various flexible substrates needs to be examined. Furthermore, future investigation on the gas sensing performance of *α*-MoO_3_ and MoS_2_ given mechanically bent and stretched conditions is needed in the future investigation. Such advancement may develop suitable state-of-the-art integration methodologies and a general guideline. Finally, there is still insufficient understanding of the sensing mechanism despite the availability of many proposed mechanisms previously reported. Working with computational simulation and modeling could help develop advanced knowledge of how gas molecules behave when exposed to sensing materials. This can help design and optimize the next generation of gas sensing materials. A similar effort is needed in examining MoSe_2_, MoTe_2_, Mo_2_C, MoC, or any other molybdenum-based materials aiming to provide more sensing material choices for a particular application. For example, molybdenum carbides are relatively more suitable for gas sensors working at higher temperatures and severe environments due to greater stability and higher melting points. Overall, molybdenum-based gas sensors hold multiple promising performances toward gas pollutant detections and have drawn great attention to the technological advancement of sensing devices. This review has provided a complete overview of recent strategies on optimizing gas sensing performance of molybdenum-based gas sensors and insight into the further advancement of these special groups as the next-generation sensing materials with high detection ability.
